# Seasonal dynamics of phyllosphere epiphytic microbial communities of medicinal plants in farmland environment

**DOI:** 10.3389/fpls.2023.1328586

**Published:** 2024-01-04

**Authors:** Chao He, Man Zhang, Xianen Li, Xueli He

**Affiliations:** ^1^ Institute of Medicinal Plant Development, Chinese Academy of Medical Sciences & Peking Union Medical College, Beijing, China; ^2^ College of Life Sciences, Hebei University, Baoding, China

**Keywords:** medicinal plants, phyllosphere, epiphytic microorganisms, seasonal dynamics, co-occurrence network

## Abstract

**Introduction:**

The phyllosphere of plants is inhabited by various microorganisms, which play a crucial role in plant physiological metabolism. Currently, there is limited research on the dynamic effects of species and seasons on plant phyllosphere microbial community diversity and microbial interactions.

**Methods:**

In this study, high-throughput sequencing technology was used to sequence the leaf surface parasitic microorganisms of five medicinal plants (*Bupleurum chinense*, *Atractylodes lancea*, *Salvia miltiorrhiza*, *Astragalus membranaceus*, and *Lonicera japonica*).

**Results:**

The results showed that bacteria and fungi clustered into 3,898 and 1,572 operational taxonomic units (OTUs), respectively. Compared to host species, seasons had a more significant impact on the a diversity of bacteria and fungi. The heterogeneity of phyllosphere microbial communities was greater in winter compared to summer. Key species analysis at the OTU level and Spearman correlation analysis demonstrated significant preferences in microbial interactions under plant and seasonal backgrounds. The network connections between bacterial and fungal communities significantly increased during seasonal transitions compared to connections with plants.

**Discussion:**

This study enhances our understanding of the composition and ecological roles of plant-associated microbial communities in small-scale agricultural environments. Additionally, it provides valuable insights for assessing the biodiversity of medicinal plants.

## 1 Introduction

“Phyllosphere microorganisms” refer to microorganisms that adhere to or parasitize the epidermal surface of plant leaves (Blakeman, 1981). Phyllosphere microorganisms originate from various sources, including wind, rain, insect air, soil, and seeds. (Chi et al., 2005; Vorholt, 2012; ZarraonaIndia et al., 2015). In reality, the phyllosphere is regarded as a challenging and unpredictable complex habitat, which is not favorable for microbial colonization due to its constant exposure to fluctuating temperature and humidity, as well as prolonged ultraviolet radiation. In recent years, there has been a growing body of research that has identified a significant abundance of microorganisms residing in the phyllosphere. The phyllosphere is considered to be a crucial ecosystem for microorganisms due to its extensive surface area, which encompasses up to 10^9^ km^2^ of vegetation worldwide. Additionally, it has the potential to support a vast number of microbial cells, estimated to be around 10^26^ (Vorholt, 2012).

Interactions between plants and their associated microbiomes are contingent upon the identities of the hosts (Zhu et al., 2022). The host species plays a crucial role in determining the composition and structure of microbial communities in the phyllosphere (Obrien and Lindow, 1989). Li et al. (2018) employed Illumina amplicon sequencing to investigate the microbial communities in the phyllosphere and rhizosphere of six distinct species of *Picea* spp. It was found that various plant species had distinct impacts on the diversity and composition of both phyllosphere and rhizosphere microbes. Bao et al. (2020) discovered that the community composition and diversity of phyllosphere epiphytic bacteria and fungi on urban green plants varied depending on the host species. Redford et al. (2010) conducted a study that examined the phyllosphere bacterial communities of 56 tree species. They also investigated the composition of bacterial communities in *Pinus ponderosa* at various locations. The researchers discovered significant differences in phyllosphere bacterial communities among the tree species. Interestingly, they discovered that the bacterial communities composition of *Pinus ponderosa* was similar across different sampling locations in Boulder, Colorado, USA. These results indicate that in different regions, the host species often have a greater impact on the composition of the phyllosphere microorganisms than environmental factors. The phenomenon under investigation may be attributed to the deliberate release of one or more biological signals by the host plant through leaf stomata or epidermis. These signals serve to attract beneficial microorganisms that aid in the plant’s growth or act as a defense mechanism against unfavorable conditions. It is also plausible that variations in the physiological structures of plant leaves enable them to selectively attract specific microorganisms for colonization. Therefore, it is imperative to acknowledge the significance of the relationship between epiphytic microorganisms and plants.

Season is widely acknowledged as a key determinant in shaping epiphytic microbial communities. Bao et al. observed notable fluctuations in the community compositions of phyllosphere epiphytic bacteria throughout the host plants’ growing season. Exogenous biodegradation pathways exhibited a notable increase in bacterial communities during in May. The findings from the network analysis revealed that the relationship between the bacterial community on leaf surfaces in May was more intricate compared to that in October, with a stronger negative correlation observed. Additionally, fluctuations in the abundance and diversity of epiphytes were observed across various seasons. (Bao et al., 2020; Bao et al., 2022). Jackson and Denney (2011) confirmed that there was seasonal pattern in the community composition of phyllosphere epiphytic bacteria on *Magnolia grandiflora* leaf layer throughout a year. Specifically, it was observed that the variation in the superbiotic bacterial community among different leaves collected during the same period was minimal. However, there was a significant difference in the bacterial community among leaves collected at different times. Notably, the bacterial community on leaves collected in August 2008 exhibited the greatest dissimilarity compared to other seasons. In addition, previous studies have demonstrated the seasonal dynamics of phyllosphere bacterial and fungal communities in *Populus deltoids*, *Ginkgo biloba*, *Pinus bungeana* and *Cunninghamia lanceolata*, respectively(Cordier et al., 2012; Peñuelas et al., 2012; Rastogi et al., 2012; Materatski et al., 2019). Therefore, it is imperative to consider different growing seasons when designing interactions between phyllosphere microbes and host plants.

Previous research on epiphytes in the phyllosphere of plants tends to focus primarily on pathogens that are of agricultural importance (Jain et al., 2019). The positive effects of the phyllosphere microbiome have, nonetheless, been substantiated by a growing body of research. Ritpitakphong et al. investigated the resistance of *Pseudomonas* sp. The efficacy of *Botrytis cinerea* control on the leaf surface of *Arabidopsis thaliana* was investigated. Specifically, under sterile conditions, the *Arabidopsis thaliana* variant bdg became as susceptible to bovines infection as the wild type (WT), while the *lacs2.3* mutant retained resistance. The resistance of *bdg* mutant to *Botrytis cinerea* was restored by adding washing solution of microbiome, which mainly include to *Pseudomonas* sp, cleaned from *lacs2.3* mutant leaf to *bdg* leaf.(Ritpitakphong et al., 2016). Busby et al. (2016) conducted an experiment where they introduced phyllosphere fungi to the leaves of *Populus trichocarpa*, resulting in a reduction in the severity of rust pathogen Melampsora × columbum infection. Phyllosphere nitrogen fixation has been identified as a significant contributor to biological nitrogen fixation in tropical ecosystems, as suggested by Cleveland et al. (1999). In Mediterranean woodland ecosystems, Rico et al. discovered that notably nitrogen-fixing bacterial populations were present in epiphytic bacteria of all *Quercus ilex* leaves (Rico et al., 2014). Therefore, phyllosphere epiphytes are microbial communities that possess significant functional significance. Predicting the functions of phyllosphere epiphytic microorganisms presents various opportunities to enhance the growth performance of host plants.

China possesses a rich and varied array of resources in the realm of traditional medicinal plants. Nevertheless, the natural regeneration rates of medicinal plants in the wild are generally low, and a significant number of these plants are currently facing the risk of extinction due to factors such as overharvesting, habitat loss, and anthropogenic activities. Industrialized plantations for medicinal plants have been established on a national scale in China. By the end of 2020, artificial cultivation of over 300 species of medicinal plants had been achieved, covering a planting area of approximately 600 million square meters (Wang et al., 2020). Nevertheless, cultivated medicinal plants often face challenges such as insufficient levels of active components and low rates of transplant survival. In recent years, there has been a significant increase in research focusing on the correlation between medicinal plants and microorganisms, which has garnered considerable attention (Ntemafack et al., 2021). The implementation of microbiological approaches to enhance resource conservation and promote sustainable utilization of medicinal plants has emerged as a significant area of research. According to Wu et al. (2021), endophytic bacteria have been found to enhance plant growth and development, enhance their resistance to both biotic and abiotic stresses, and stimulate the production of novel compounds that may have potential medical applications. Han et al. (2021) made the discovery that the dark septate endophyte successfully colonized all 25 medicinal plants within the farming region of northern China. Chen et al. (2011) conducted a study in which they successfully isolated and identified approximately 80 culturable endophytic fungi from 10 different species of medicinal Dendrobium. The identification process involved the use of both morphological and molecular techniques. The findings of the study revealed a significant level of biodiversity among the endophytic fungi associated with Dendrobium plants. The current body of research regarding the advantageous impacts of the phyllosphere epiphytic microbial community on medicinal plants is constrained.

To examine the influence of host plants and seasonal variations on the composition of epiphytic bacterial and fungal communities in medicinal plants within agro-ecosystems, as well as to explore the associations between epiphytes and their host plants, and the functional capabilities of epiphytes, our study was conducted at the Anguo Medicine Planting Site. The abundance, diversity, and composition of phyllosphere epiphytic bacterial and fungal communities of 5 medicinal plants, viz., *Bupleurum chinense* DC., *Atractylodes lancea* (Thunb.) DC, *Salvia miltiorrhiza* Bge., *Astragalus membranaceus* (Fisch.) Bge., *Lonicera japonica* Thunb., was conducted using Illumina Miseq high-throughput sequencing (HTS) technology. Additionally, co-occurrence networks of microorganisms between plants and seasons were established. We formulated the following hypotheses: (1) The phyllosphere of various medicinal plants harbors diverse and distinct bacterial and fungal communities. (2) The composition of epiphytic communities varies based on the identity of the plant or the season. (3) Extensive intra-community interactions occur among epiphytes within the same season or plant. This research will lay the groundwork for revealing the ecological significance and functions of epiphytic communities in agricultural ecosystems, as well as the biodiversity and survival strategies of epiphytic communities in various environments.

## 2 Materials and methods

### 2.1 Study site and sample collection

The sampling sites were situated in the Anguo Medicine Planting Site (38°42′ N, 115°32′ E) within Hebei Province, China. The study area exhibits a characteristic temperate continental climate, characterized by an average monthly temperature of 12.3°C and a precipitation level of 51.6 mm. Cultivated medicinal plants at the planting site underwent two rounds of irrigation throughout their entire growing season. In addition, water-soluble fertilizer is used once a year to irrigate the roots of all plants. The amount of fertilization per time was N 180 kg/hm^2^, P_2_O_5_ 90 kg/hm^2^, K_2_O 180 kg/hm^2^.In June and November 2021, leaf samples were gathered from five distinct species of medicinal plants, specifically *Bupleurum chinense*, *Atractylodes lancea*, *Salvia miltiorrhiza*, *Astragalus membranaceus*, and *Lonicera japonica*. Three replicated plots were designated for each species, and within each plot, three healthy plant individuals were randomly chosen, ensuring a minimum distance of 50 meters between each selection. A minimum of 100 grams of fresh and healthy leaves were harvested from each plant at a consistent height above the ground, utilizing sterile scissors. Leaf samples were promptly placed into ice boxes at a temperature of 4°C and subsequently transported to the laboratory. Subsamples of three plant individuals were collected from each plot and combined into a single sample for the purpose of extracting epiphytes.

### 2.2 Phyllosphere epiphytic microbial isolation

Five grams of plant leaves were weighed and put into a 50 ml centrifuge tube, with 50 mL of 0.1 M potassium phosphate buffer (PPB, pH=8.0) added. The leaf sample in tubes were washed with 1 min sonication and 10 s vortex, and repeated. Then the leaves were transferred to new tubes with 50mL of 0.1M PPB and wash again. The suspension from two washes were mixed and filtered through a 0.2µm membrane., The filter membranes with epiphytes were snap frozen in liquid nitrogen and stored at -80°C in refrigerator (Bodenhausen et al., 2013).

### 2.3 Sample DNA extraction

The genomic DNA from phyllosphere epiphytic microorganisms was extracted from filter membranes using the FastDNA® Spin Kit for Soil (MP Biomedicals, USA) according to user’s manual. The DNA purity and concentration were measured with a NanoDrop 2000 spectrophotometer (Thermo Fisher Scientific, USA), and DNA integrity was examined using 1% agarose gel electrophoresis.

### 2.4 PCR amplification and library creation for sequencing

The 16S V3-V4 region of epiphytic bacteria and ITS1 region of epiphytic fungi were amplified with 338F/806R (ACTCCTACGGGAGGCAGCAG/GGACTACHVGGGTWTCTAAT) and ITS1F/ITS2 (CTTGGTCATTTAGAGGAAGTAA/GCTGCGTTCTTCATCGATGC), respectively, with ABI GeneAmp® 9700 PCR thermocycler (ABI, USA). PCR reactions were performed in a 20 µL system, which included 2 µL 10× Buffer, 2 µL 2.5 mM dNTPs, 0.8 µl each of 5 µM primers, 0.2 µL TaqPolymerase, 0.2 µL BSA, 10 ng template DNA, and ddH_2_O supplemented to 20 µL. The amplification for 16S V3-V4 region of bacteria were performed under following conditions: denaturation at 95°C for 3 min; 95°C for 30 s, 55°C for 30 s, 72°C for 45 s, 27 cycles; extension at 72°C for 10 min. For ITS1 region of fungi, PCR amplifications were conducted with same reaction system and condition, except the reaction were repeated for 35 cycles. Each amplification was replicated for three times. The replicated PCR products of a same sample were pooled, recovered using 2% agarose gel, and further purified using the AxyPrep DNA Gel Extraction Kit (Axygen Biosciences, Union City, CA, USA). The recovered PCR products were then quantified using a Quantus™ Fluorometer (Promega, USA). The purified amplification fragments were mixed in equal amounts, and the libraries were constructed using the NEXTFLEX® Rapid DNA-Seq Kit. Shanghai Majorbio Bio-pharm Technology Co., Ltd used Illumina’s MiSeqPE300 platform to carry out the final sequencing. The raw data were deposited in NCBI SRA database (PRJNA942029, PRJNA942068).

### 2.5 Data processing

The paired-ended raw sequences were spliced and quality controlled using software tools *fastp* (version 0.19.6) and FLASH (version 1.2. 11) with following steps: (1) filter the bases with quality values below 20 and reads containing N bases, set a window of 50 bp, truncate the bases if the average quality value within window is below 20, and finally filter the reads below 50 bp after quality control; (2) pairs of reads were spliced in accordance with the overlap between PE reads, with a minimum overlap length of 10 bp; (3) a maximum mismatch ratio of 0.2 was permitted in the overlap area of the spliced sequence, and non-conforming sequences were removed; (4) samples were demultiplexed based on the barcode. The quality controlled spliced sequences were clustered into operational taxonomic units (OTUs) based on 97% similarity using UPARSE software (version 7.1). All sequences with mitochondrial and chloroplast annotations were stripped off. The samples were rarefied due to the minimum sum of sequences in all samples to reduce the effect of sequencing depth on the subsequent analysis of alpha- and beta-diversity data. The taxonomic placement of epiphytic bacteria and fungi were annotated according to Silva 16S rRNA gene database (v 138) and UNITE databases (Version 8.0), respectively, using the RDP classifier (version 2.11) with 70% confidence threshold. The community composition for each sample was analyzed at various species taxonomic levels. The BugBase, the FAPROTAX (Louca et al., 2016) manual construction database, and the FUNGuild (Fungi Functional Guild) database were used to perform bacterial phenotypic predictions, functional predictions, and ecological functional predictions, respectively. Relative abundance is the percentage of abundance of a species in a community that is the sum of the abundance of all species, all references below are to RA.

### 2.6 Statistical analysis

The Majorbio Bio Cloud platform (https://cloud.majorbio.com) was used for all data analysis. Alpha diversity indices were estimated using Wilcoxon rank sum test for inter-group variance analysis of alpha-diversity *via* the mothur software (Schloss et al., 2009). Non-metric multidimensional scaling (NMDS) based on the bray-curtis distance algorithm, was used to examine the similarity of microbial community structure between samples, and PERMANOVA non-parametric test was used to test the significance of differences in microbial community structure between groups.

Network analysis was used to predict patterns of interaction between phyllosphere epiphytic bacteria and fungi. Spearman correlation tests were performed, and networks were constructed using OTU based on the top 50 of abundance. The total abundance of the top 50 OTUs of bacteria is 40.8%, while that of fungi is 43.6%. Correlations with absolute values of correlation coefficient (rho) greater than 0.8 and p-values less than 0.01 were retained for network analysis (Wang et al., 2017). Co-occurrence networks were analyzed using Cytoscape (version 3.8.2), and the number of nodes and edges, clustering coefficients and network density were analyzed using the built-in application network analyzer (Shannon et al., 2003; Assenov et al., 2008). In addition, modules and highly interconnected nodes and central taxa, were analyzed using MCODE (Bader and Hogue, 2003). Sobs refers to observed richness. Shannon is one of the indices used to estimate the diversity of microorganisms in a sample, with higher Shannon values indicating higher community diversity. Shannoneven is a measure of homogeneity based on the Shannon index. The Student’s T test, uses t-distribution theory to infer the probability of a difference occurring and thus compare whether the difference between two means is significant. The Kruskal-Wallis H test, is a method of extending the Wilcox rank sum test for two independent samples to a non-parametric test for multiple (≥ 3) independent samples.

## 3 Results

### 3.1 Epiphytic microbial community composition

A total of 1,272,729 reads were obtained for bacterial sequences, while 1,900,620 reads were obtained for fungal sequences, using high-throughput sequencing. After implementing quality control measures to eliminate low-quality sequences, a total of 1,189,582 reads for bacterial sequences and 1,752,919 reads for fungal sequences were obtained, meeting the necessary criteria for further analysis. The sequences in both datasets were clustered, resulting in a total of 3,537 bacterial OTUs and 1,450 fungal OTUs, respectively. The rarefaction curves of the Sobs indices for phyllosphere epiphytic bacteria and fungi at the OTU level demonstrated a plateau phenomenon as the number of sampled reads increased. This observation suggests that the sampling and sequencing strategy utilized in this study were adequate for conducting diversity analysis (**Supplementary Figure S1**).

A comprehensive analysis of phyllosphere epiphytic bacteria from five medicinal plants during both summer and winter seasons resulted in the identification of a total of 3,537 OTUs. These OTUs belonged to 38 different phyla, 101 classes, 232 orders, 390 families, 859 genera, and 1,601 species. Meanwhile, the assemblage of phyllosphere epiphytic fungi consisted of a total of 1,450 OTUs, which encompassed 9 phyla, 34 classes, 89 orders, 205 families, 437 genera, and 780 species.

**Figure 1:**
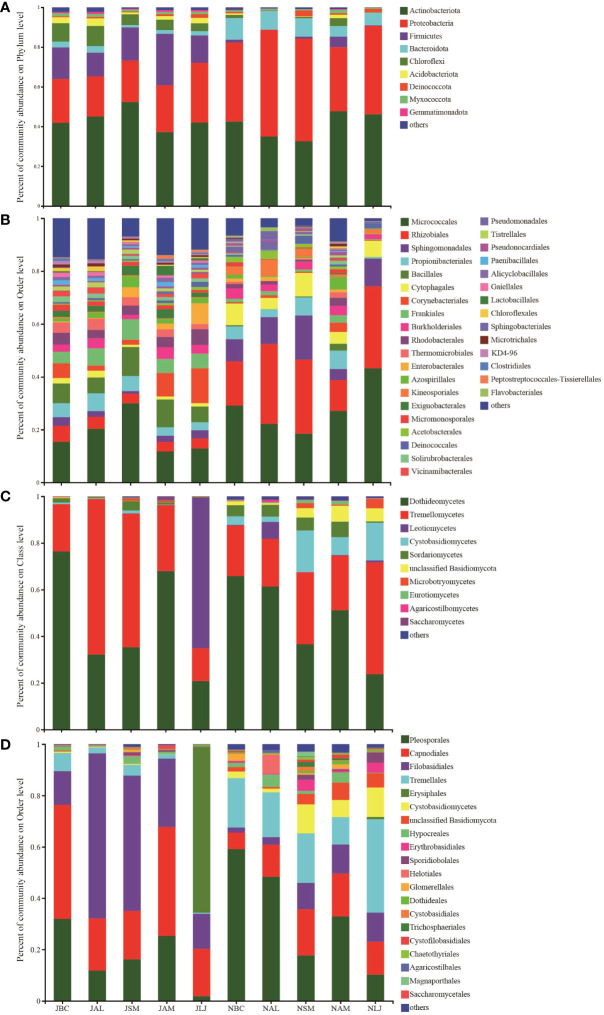
Relative abundance of epiphytic bacterial **(A, B)** and fungi **(C, D)** in medicinal plants.Represents < 0.01% of the total reads of epiphytic bacterial and fungi were all assigned to “Others”. J, June-Summer; N, November-Winter; BC, *Bupleurum chinense*; AL, *Atractylodes lancea*; SM, *Salvia miltiorrhiza*; AM, *Astragalus membranaceus*; LJ, *Lonicera japonica*.

The composition of phyllosphere epiphytic bacteria at the phylum level (**Figure 1A**) exhibited two predominant phyla during both summer and winter across the five plants studied. These phyla were Actinobacteriota (Relative Abundance=32.61%-52.40%) and Proteobacteria (RA=20.28%-53.70%). The Firmicutes group exhibited the third highest abundance during the summer months, with a relative abundance ranging from 11.89% to 25.94%. However, their presence significantly decreased in November, with a relative abundance ranging from 0.04% to 5.34%. In contrast, Bacteroidota (RA=5.37%-10.94%) emerged as the third most prevalent group during the winter season across all plant species. The order Micrococcales (RA=11.73%-43.22%) was found to be the dominant epiphytic bacterial community in both summer and winter across all host species (**Figure 1B**). The Rhizobiales taxonomic group exhibited a seasonal turnover, with relative abundance (RA) ranging from 3.62% to 6.09% in summer, and significantly higher values of 11.77% to 31.03% in winter.

In both summer and winter, the composition of phyllosphere epiphytic fungal communities in host plants was found to be similar at the phylum level (**Figure 1C**). The dominant phyla in these communities were Dothideomycetes (RA=23.73%-76.40%) and Tremellomycetes (RA=20.20%-66.69%). An exception was observed in the summer community on L. maackii, where Leotiomycetes exhibited predominance with a relative abundance (RA) of 64.58%. In contrast, Dothideomycetes and Tremellomycetes had RAs of 20.81% and 14.09%, respectively. At the order level (**Figure 1D**), the fungal communities of all host plants in summer, with the exception of L. maackii, were dominated by Filobasidiales (RA=13.12% - 64.33%), Capnodiales (RA=18.82% - 44.44%), and Pleosporales (RA=11.79% - 31.95%). The fungal communities associated with L. maackii during the summer season were found to be predominantly composed of Erysiphales (RA=64.56%), Filobasidiales (RA=13.62%) and Capnodiales (RA=18.46%). Meanwhile, during the winter season, the Tremellales (RA=10.64% - 36.42%), along with the Pleosporales (RA = 10.22% - 59.11%) and Capnodiales (RA = 6.42% - 17.99%), exhibited dominance in the epiphytic fungal communities.

**Figure 2:**
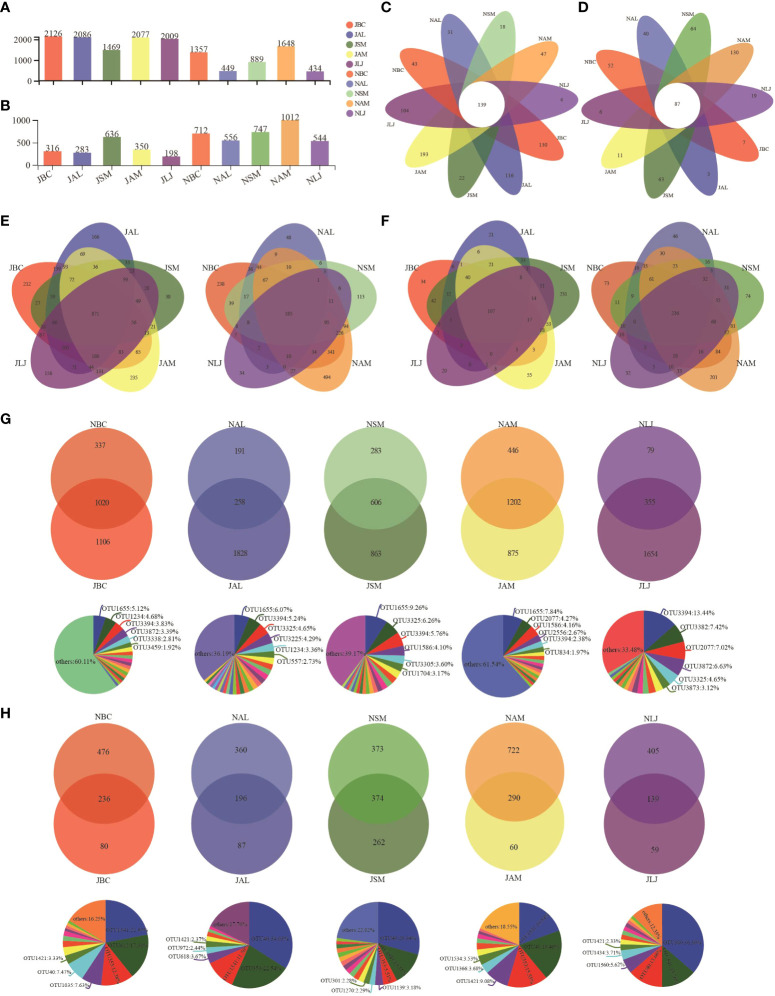
Distribution of the epiphytic bacterial **(A, C, E, G)** and fungi **(B, D, F, H)** OTUs in different plants **(E, F)** and in different seasons **(G, H)**. J, June-Summer; N, November-Winter; BC, *Bupleurum chinense*; AL, *Atractylodes lancea*; SM: *Salvia miltiorrhiza*; AM, *Astragalus membranaceus*; LJ, *Lonicera japonica*.

The study revealed that the prevalence of bacterial OTUs was significantly greater during the summer in comparison to the winter across all host plants (**Figure 2A**). During the summer, B. chinense demonstrates the highest OTU richness among the species, whereas *S. miltiorrhiza* displays the lowest OTU richness. In the winter, the plant species *A. membranaceus* displays the highest level of abundance, whereas *L. japonica* exhibits the lowest level of abundance. A comprehensive analysis revealed that a total of 139 bacterial OTUs exhibited consistent occurrence across all host plants and throughout both seasons, as illustrated in **Figure 2C**. When analyzing the data with respect to seasons, it was observed that there were 871 bacterial OTUs were present during the summer, whereas only 185 were detected during the winter (**Figure 2E**). In the summer, the number of host-specific OTUs observed among different plant species. The species *S.miltiorrhiza* exhibited the lowest number of OTUs, with a count of 38, whereas *A. membranaceus* had the highest number of OTUs, totaling 235. *Lonicera japonica* demonstrated the lowest count of distinct OTUs, amounting to 34, while *A.membranaceus* displayed the highest count of unique OTUs, reaching 494 during the winter (**Figure 2E**). The summer and winter communities of five host species, namely *B.chinense*, *A.lancea*, *S.miltiorrhiza*, *A. membranaceus*, and *L. japonica*, displayed shared OTUs of 1,020, 258, 606, 1,202, and 355, respectively (**Figure 2G**). Pie charts were employed to visually depict the distribution of OTUs within the intersecting subsets of a particular plant species during both the summer and winter (**Figure 2G**). We have observed that different plant species display diverse bacterial OTU throughout various seasons.

The abundance of epiphytic fungi OTUs in winter is significantly higher than in summer, in contrast to the phenomenon observed in bacteria. In the summer, *S. miltiorrhiza* exhibits the highest OTU abundance, while *L. japonica* has the lowest. In winter, A. membranaceus exhibits the highest OTU abundance, while *L. japonica* shows the lowest (**Figure 2B**). Analysis of the petal diagram reveals a total of 87 OTUs across all plant species (**Figure 2D**). In the analysis of season-specific OTUs, the results indicate that during the summer, *S. miltiorrhiza* has the highest number of specific OTUs, while *L. japonica* has the lowest. In winter, *A. membranaceus* has the highest number of specific OTUs (**Figure 2F**). In summer and winter, *B. chinense*, *A. lancea*, *S. miltiorrhiza*, *A. membranaceus*, and *L. japonica* have 236, 196, 374, 290, and 139 shared OTUs, respectively (**Figure 2H**).

### 3.2 Epiphytic microbial alpha-diversity

**Figure 3:**
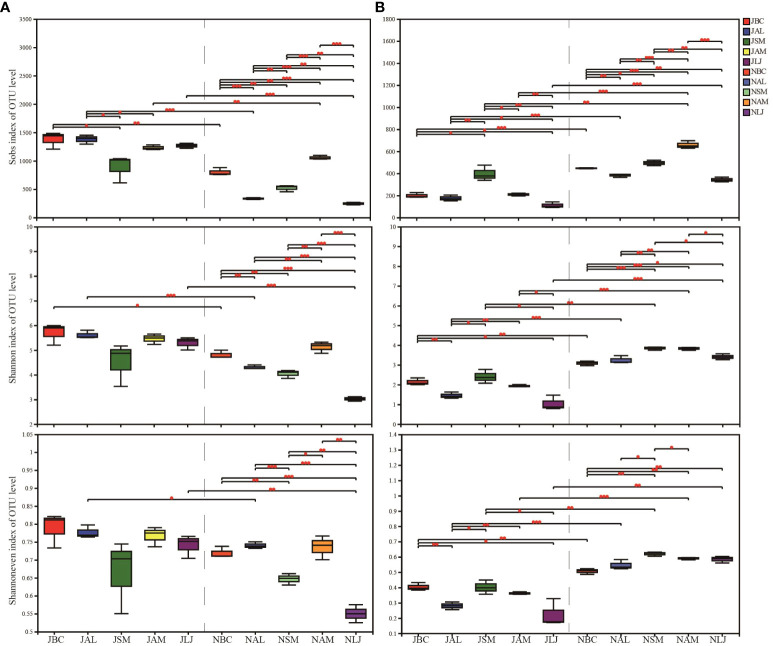
OTU richness, Shannon and Shannoneven of phyllospheric epiphytic bacterial **(A)** and fungi **(B)**. * P < 0.05, ** P < 0.01 and *** P < 0.001. J, June-Summer; N, November-Winter; BC, *Bupleurum chinense*; AL, *Atractylodes lancea*; SM, *Salvia miltiorrhiza*; AM, *Astragalus membranaceus*; LJ, *Lonicera japonica*.

The alpha diversity of epiphyte OTUs was assessed based on host plants and seasons using Sobs, Shannon, and Shannoneven indices. In the context of bacterial communities, we have observed a lesser degree of variation in diversity among summer communities. Significant differences were observed between *B. chinense* and *S. miltiorrhiza*, *A. lancea* and *S. miltiorrhiza*, and *A. lancea* and *A. membranaceus*, as indicated by the Sobs index. No significant disparity in host plant selection during the summer was observed by Shannon and Shannoneven indices. However, a significant influence of the host was observed on the diversity of epiphytic bacteria, as evidenced by the diversity indices which showed statistically significant differences (p < 0.05) among plants during the winter (**Figure 3A**). *B. chinense*, *A. lancea*, and *L. japonica* exhibited notable variations in bacterial diversity between the summer and winter, as indicated by the Shannon index.

In the fungal communities, notable variations were observed in the Sobs index, Shannon index, and Shannoneven index, both among the host plants during the summer and among the host plants during the winter (**Figure 3B**). Furthermore, *B. chinense*, *A. lancea*, *S. miltiorrhiza*, and *L. japonica* exhibited notable variations in fungal populations across seasons, as indicated by the diversity indices.

### 3.3 Comparison of the similarity of phyllosphere epiphytic microbial communities

**Figure 4:**
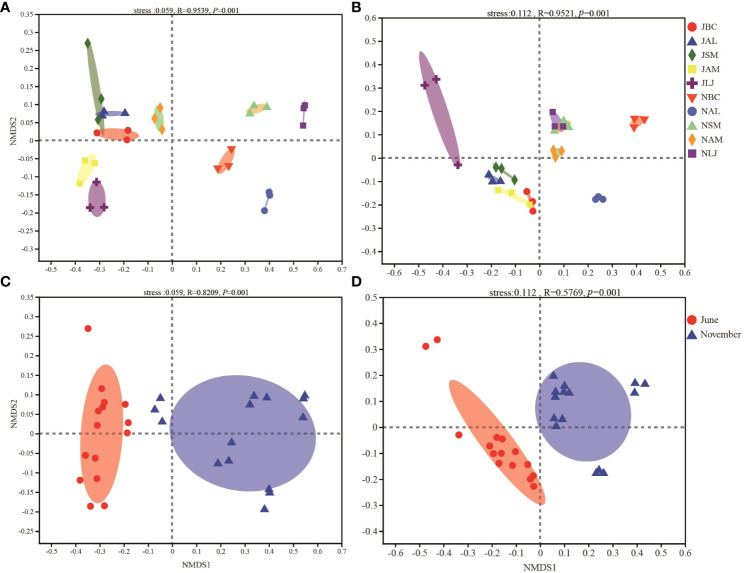
Nonmetric multidimensional scaling (NMDS) ordination of the bacterial **(A, C)** and fungi **(B, D)** community composition among different plant species and seasons. J, June-Summer; N, November-Winter; BC, *Bupleurum chinense*; AL, *Atractylodes lancea*; SM, *Salvia miltiorrhiza*; AM, *Astragalus membranaceus*; LJ, *Lonicera japonica*.

The composition of the epiphytic bacterial community exhibited significant variations among different plants (F=0.9639, P=0.001) and across different seasons (F=0.8209, P=0.001), as determined by NMDS and ANOSIM tests (**Figures 4A, C**). The PERMANOVA analysis demonstrated that plant species accounted for 85.42% of the variation in the composition of the phyllosphere epiphytic bacterial community (P=0.001), while seasons explained 43.89% of the variation (P=0.001) for seasons (**Table 1**).

**Table 1:** Effects of plant species and seasons on microbial community structure based on PERMANOVA.

	Bacterial		Fungi	
	*R^2^* (%)	Pr(>*F*)	*R^2^* (%)	Pr(>*F*)
Plant species	85.41	0.001	91.24	0.001
Season	43.89	0.001	29.87	0.001

The ANOSIM results indicated that the community composition of phyllosphere epiphytic fungi was significantly influenced by both seasons (F=0.5769, P=0.001) and plants (F=0.9521, P=0.001) (**Figures 4B, D**). The composition of the epiphytic fungal community was significantly influenced by the plant species (91.24%, P=0.001) and the season (29.87%, P=0.001), as determined by PERMANOVA analysis (**Table 1**).

### 3.4 Analysis of differential phyllosphere epiphytic microbial communities

**Figure 5:**
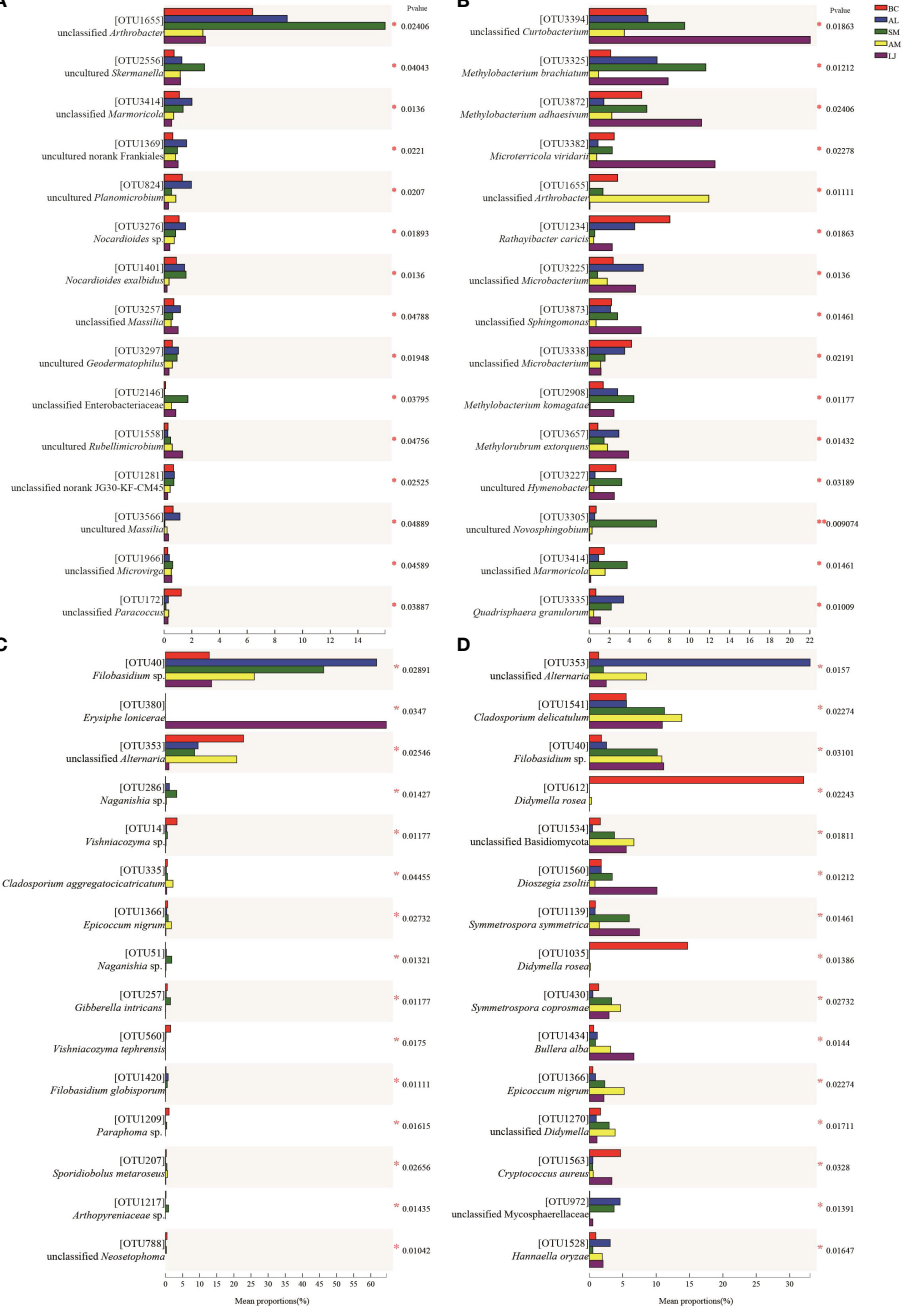
Anosim tests the richness differences of the 15 most abundant in phyllospheric epiphytic bacterial **(A, B)** and fungal **(C, D)** communities between different plant species in summer **(A, C)** and winter **(B, D)**. BC, *Bupleurum chinense*; AL, *Atractylodes lancea*; SM, *Salvia miltiorrhiza*; AM, *Astragalus membranaceus*; LJ, *Lonicera japonica*. *indicates the significant difference at P < 0.05. **indicates the significance difference at P < 0.01.

The colonization patterns of epiphytic bacterial and fungal taxa exhibited notable variations across different host plants in our study. ANOSIM tests conducted among various plant species revealed significant differences in the abundance of 15 most prevalent taxa in both summer and winter communities (**Figure 5**). In the summer, the unclassified *Paracoccus* (OTU172) exhibited a significant increase in abundance within *B. chinense*. Unclassified *Marmoricola* (OTU3414), uncultured *Frankiales* (OTU1369), uncultured *Planomicrobium* (OTU824), and *Nocardioides* sp. were identified in the sample. (OTU3276) exhibited a significant enrichment in *A. lancea*. Unclassified *Arthrobacter* (OTU1655), uncultured *Skermanella* (OTU2556), and unclassified *Enterobacteriaceae* (OTU2146) exhibited significant enrichment in *S. miltiorrhiza*. The presence of Uncultured *Rubellimicrobium* (OTU1558) was significantly higher in *L.japonica* (**Figure 5A**). In the winter, the *A. lancea* species exhibits a significantly higher abundance of unclassified *Microbacterium* (OTU3225) and *Quadrisphaera granulorum* (OTU3335). *S. miltiorrhiza* was found to be colonized by *Methylobacterium brachiatum *(OTU3325), *Methylobacterium komagatae* (OTU 2908), and an uncultured *Novosphingobium* (OTU3305), which were present in high abundance. The presence of unclassified *Arthrobacter* (OTU1655) was significantly higher in *A. membranaceus*. Unclassified *Curtobacterium* (OTU 3394), *Methylobacterium adhaesivum* (OTU 3872), *Microterricola viridarii* (OTU3382), unclassified *Sphingomonas* (OTU3873), and *Methylorubrum extorquens* (OTU3657) exhibited significant differences in their presence within *L. japonica* (**Figure 5B**).

Similarly, the colonization patterns of phyllosphere epiphytic fungi with high abundance varied significantly across different host plants. In the summer, we discovered the presence of unclassified Alternaria (OTU353), *Vishniacozyma* sp. (OTU14), *Vishniacozyma tephrensis*, and *Paraphoma* sp. (OTU1209) exhibited enrichment in *B. chinense*. *Filobasidium* sp. (OTU40) and *Filobasidium globisporum* (OTU1420) exhibited higher abundance in *A. lancea*. *Naganishia* sp. (OTU286, OTU51) and *Gibberella intricans* (OTU257) were found to be enriched in *S. miltiorrhiza*. *Cladosporium aggregatocicatricatum *(OTU335) and *Epicoccum nigrum *(OTU1366) were found to be abundant in *A. membranaceus*. *Erysiphe lonicerae*(OTU380) was found to be abundant in *L. japonica* (**Figure 5C**). In the winter, there was a notable increase in the presence of *Didymella rosea *(OTU1035) in *B. chinense*. *A. lancea* was colonized by enriched *unclassified Alternaria* (OTU353). *Cladosporium delicatulum* (OTU1541), an unclassified *Basidiomycota* (OTU1534), *Symmetrospora coprosmae* (OTU430), *Epicoccum nigrum* (OTU1366), and an unclassified *Didymella* (OTU1270) exhibited higher abundance in *A. membranaceus*. *Filobasidium* sp. (OTU40), *Dioszegia zsoltii* (OTU1560), *Symmetrospora symmetrica*(OTU1139), and *Bullera alba* (OTU1434), exhibited a significant enrichment in *L. japonica* (**Figure 5D**).

**Figure 6:**
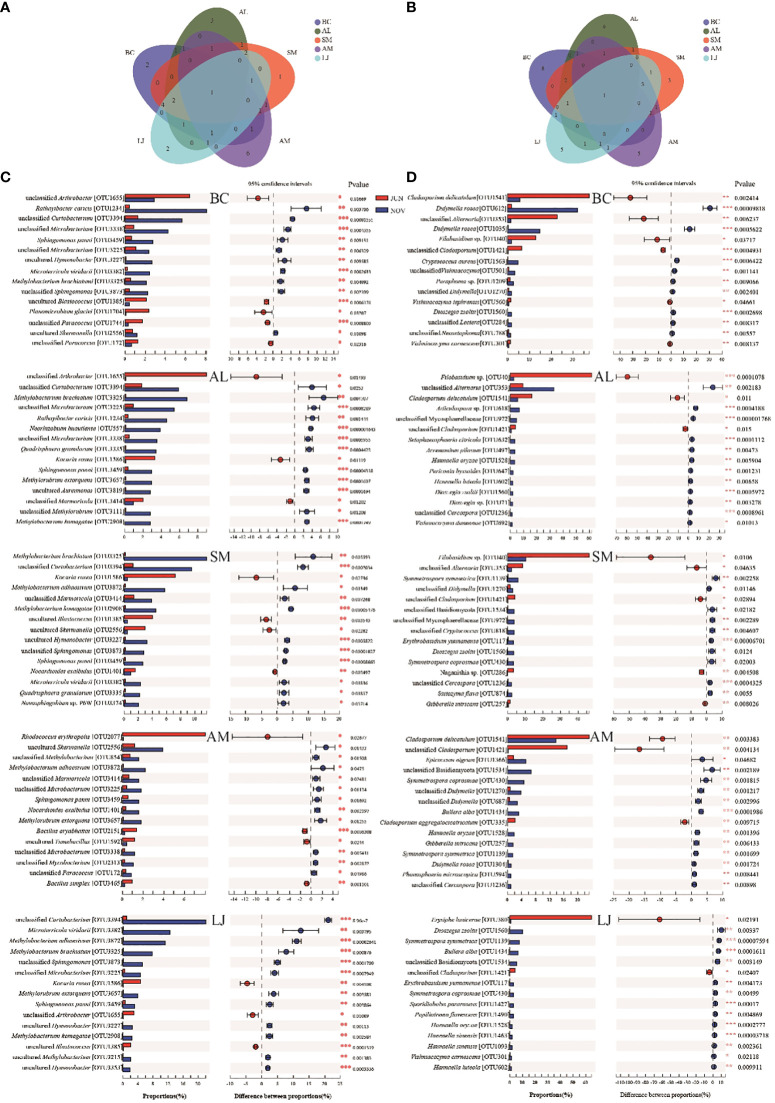
The distribution of the 15 most abundant epiphytic bacterial **(A, C)** and fungal **(B, D)** species in phyllospheric of different plant species in summer and winter. The * symbol indicates the significant difference P < 0.05. The ** symbol indicates the significance level intervenes between P < 0.01. The *** symbol indicates difference is very significant, P < 0.001.

Significant variations in colonization patterns were observed between seasons for both bacterial and fungal taxa in our study. Through the implementation of Veen analysis to assess the diversity of epiphytic bacteria and fungi across various plant species, our findings indicate that there is only one shared bacterial species among all the species examined. However, there are distinct bacterial species that are unique to each of the following plant species: *A.lancea* (3 unique species), *S.miltiorrhiza* (1 unique species), *A.membranaceus* (6 unique species), *L.japonica* (2 unique species), and *B.chinense* (2 unique species). Among fungi, the total number of species shared by all species is 1, whereas *A. lancea*, *S.miltiorrhiza*, *A. membranaceus*, *L. japonica*, and *B. chinense* have 6, 3, 5, 5, and 8 unique species, respectively(**Figures 6A, B**).

Specifically, we found that the 15 most abundant taxa were depicted separately based on the host plants (**Figure 6C**). Of these 15 bacterial taxa, only *Sphingomonas panni* (OTU3459) occurs in all host plants. *A. membranaceus* has the most uniquely different bacterial taxa, including *Rhodococcus erythropolis* (OTU2077),unclassified *Methylobacterium* (OTU854), *Bacillus aryabhattai* (OTU2151), uncultured *Tumebacillus* (OTU 1592), unclassified *Mycobacterium* (OTU2313) and *Bacillus simplex *(OTU3465). *S. miltiorrhiza* exhibited the lowest number of distinct bacterial taxa, with only one identified as *Novosphingobium* sp. P6W(OTU3374).

Among the 15 abundant fungal taxa with seasonal differentiation in each plant species, only one taxon, viz., unclassified *Cladosporium* (OTU1421), was presented among all host plants (**Figure 6D**). *B. chinense* exhibits a high abundance of distinctive fungal taxa, such as *Didymella rosea* (OTU1035),*Cryptococcus aureus* (OTU1563), unclassified *Vishniacozyma* (OTU501), *Paraphoma* sp. (OTU1209), *Vishniacozyma tephrensis* (OTU560), unclassified *Lectera* (OTU284), unclassified *Neosetophoma* (OTU788) and *Vishniacozyma carnescens* (OTU301). *S. miltiorrhiza* exhibits the lowest number of distinct fungal taxa, including unclassified *Cryptococcus* (OTU818), *Naganishia* sp. (OTU286), and *Saitozyma flava* (OTU874).

### 3.5 Correlation network analysis

**Figure 7:**
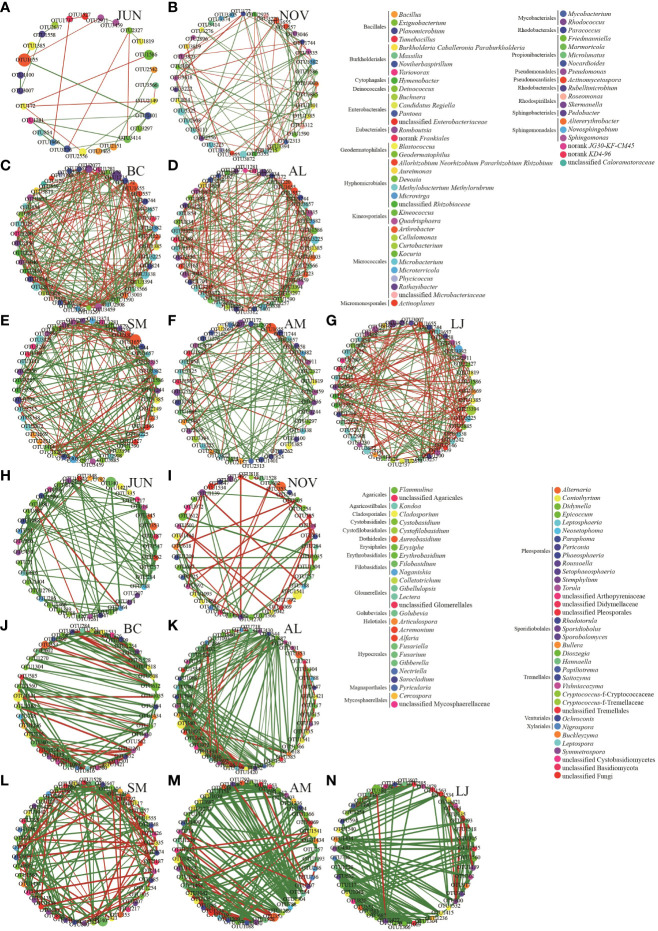
Co-occurrence networks of microbial taxa in the bacterial **(A–G)** and fungi **(H–N)** communities.Note: Nodes represent bacterial OTUs, different node colors are used to distinguish different bacterial genus, MCODE Rank is a module with significant relationships calculated based on networks between plants, and the relative abundance of OTU is represented by the node size. Edges represent significant interaction between OTUs, green edges indicate a positive correlation, red edges indicate negative correlation, and the width of each edge reflects the Spearman correlation coefficient between nodes.

Correlation network analysis using Spearman correlation was conducted to examine the variations in network structures of epiphytic communities among different plants and across seasons. This analysis was based on the abundance of the top 50 most abundant OTUs (**Figure 7**). The networks of communities consisting of epiphytic bacteria from all five host plants exhibited a decrease in the number of nodes, edges, and clustering coefficients during the summer compared to the winter. Conversely, the network densities and centrality coefficients were higher in the summer than in the winter (**Figures 7A, B**; **Table 2**). The epiphytic bacterial community networks within a specific host exhibited distinct network characteristics across different seasons (**Figures 7C–G**; **Table 2**). For instance, the bacterial communities in *B. chinense* exhibited the highest number of edges, network density, and centrality coefficient. On the other hand, *A. lancea* had the highest number of nodes but the lowest clustering coefficient. *S.miltiorrhiza*, in contrast, had the lowest centrality coefficient. *A. membranaceus* had the fewest nodes, edges, and lowest network density. Lastly, *L. japonica* displayed the highest clustering coefficient.

**Table 2:** Structural attributes of networks of phyllospheric epiphytic bacteria(B) and fungi(F) for plant species and seasons.

		Jun	Nov	JBC-NBC	JAL-NAL	JSM-NSM	JAM-NAM	JLJ-NLJ
Number of nodes	B	27	39	45	48	46	42	47
	F	41	42	43	47	47	47	46
Number of edges	B	26	84	225	189	178	120	187
	F	95	59	141	204	141	186	185
+	B	16	52	123	93	119	96	88
	F	92	35	99	162	89	148	168
_	B	10	32	102	96	59	24	99
	F	3	24	42	42	52	38	17
Avg. number of neighbors	B	2.364	4.629	10.419	7.875	8.045	5.950	8.267
	F	6.087	3.067	6.558	8.681	6.222	7.915	8.043
Network diameter	B	6	13	6	8	7	10	7
	F	6	8	10	7	6	5	7
Characteristic path length	B	2.636	4.328	2.316	2.874	2.807	4.050	2.995
	F	2.419	3.124	3.815	2.898	3.083	2.744	2.923
Clustering coefficient	B	0.212	0.531	0.627	0.577	0.630	0.600	0.663
	F	0.538	0.331	0.628	0.656	0.625	0.507	0.646
Network density	B	0.236	0.136	0.248	0.168	0.187	0.153	0.188
	F	0.277	0.219	0.156	0.189	0.141	0.172	0.179
Network heterogeneity	B	0.487	0.453	0.413	0.41	0.409	0.548	0.412
	F	0.539	0.577	0.392	0.538	0.293	0.345	0.377
Network centralization	B	0.200	0.136	0.189	0.136	0.121	0.163	0.136
	F	0.344	0.242	0.111	0.212	0.137	0.138	0.162
Connected components	B	5	2	2	1	2	2	2
	F	4	7	1	1	2	1	1
Analysis time (sec)	B	0.047	0.047	0.047	0.050	0.043	0.0447	0.031
	F	0.063	0.043	0.032	0.053	0.043	0.046	0.037

Community networks comprising epiphytic fungi from all five host plants exhibited higher edge, clustering coefficients, network density, and centrality coefficients during the summer compared to the winter (**Figures 7H, I**; **Table 2**). The network characteristics of epiphytic fungal communities associated with a specific host exhibited variations across different seasons (**Figures 7J–N**, **Table 2**). Among the studied plant species, *B. chinense* exhibited the lowest number of nodes, edges, and centrality coefficients. On the other hand, *A. lancea* displayed the highest number of nodes, edges, clustering coefficients, and network density. *S. miltiorrhiza* had the highest number of nodes, the fewest edges, and the lowest network density. *A. membranaceus* had the highest number of nodes and the fewest clustering coefficients. Lastly, *L. japonica* exhibited the highest number of centrality coefficients.

Both positive and negative correlations were observed in the epiphytic bacterial and fungal networks in our study (**Figure 7**). The community who possessed advanced degrees were identified as keystone taxa within the network (**Table 3**). In addition, it is evident that negative correlations have significantly increased within the fungal winter community. In our study, we observed predominantly positive associations between taxa in fungal communities across seasons in all five host plants (**Figures 7J–N**).

**Table 3:** The ten keystone species in phyllospheric epiphytic bacteria networks for plant species and seasons.

	Order	Genus	OTU	degree	+	-	Abundance (%)
Jun	Propionibacteriales	*Marmoricola*	OTU3414	4	3	1	3.91
	Propionibacteriales	*Nocardioides*	OTU1401	4	4	0	3.09
	Frankiales	*Geodermatophilus*	OTU2327	3	1	2	1.50
	Micrococcales	*Arthrobacter*	OTU1655	3	2	1	25.46
	Rhodobacterales	*Paracoccus*	OTU172	3	0	3	1.56
	Thermomicrobiales	norank JG30-KF-CM45	OTU1281	3	3	0	1.92
	Propionibacteriales	*Nocardioides*	OTU3276	3	2	1	3.15
	Frankiales	*Blastococcus*	OTU2149	3	1	2	1.87
	Sphingomonadales	*Sphingomonas*	OTU2911	2	1	1	1.50
	norank KD4-96	norank KD4-96	OTU1737	2	0	2	1.44
Nov	Azospirillales	*Skermanella*	OTU2556	9	6	3	1.98
	Rhodobacterales	*Paracoccus*	OTU1744	8	3	5	0.70
	Micrococcales	*Arthrobacter*	OTU1655	7	4	3	5.47
	Cytophagales	*Hymenobacter*	OTU2975	7	3	4	1.00
	Rhodobacterales	*Paracoccus*	OTU172	7	4	3	0.74
	Sphingomonadales	*Sphingomonas*	OTU3873	7	2	5	4.39
	Cytophagales	*Hymenobacter*	OTU3353	7	4	3	1.30
	Corynebacteriales	*Mycobacterium*	OTU2313	7	3	4	0.58
	Frankiales	*Blastococcus*	OTU1385	7	6	1	0.85
	Micrococcales	*Microterricola*	OTU3382	6	3	3	6.39
JBC-NBC	Corynebacteriales	*Rhodococcus*	OTU2077	18	2	16	4.70
	Micrococcales	*Curtobacterium*	OTU3394	17	11	6	6.81
	Micrococcales	*Microterricola*	OTU3382	16	10	6	2.71
	Rhodobacterales	*Paracoccus*	OTU172	16	5	11	1.83
	Cytophagales	*Hymenobacter*	OTU3227	16	10	6	2.71
	Cytophagales	*Hymenobacter*	OTU3356	16	10	6	0.73
	Rhizobiales	*Methylorubrum*	OTU3872	16	10	6	6.01
	Rhizobiales	*Methylorubrum*	OTU2908	16	10	6	1.38
	Burkholderiales	*Variovorax*	OTU3260	15	9	6	1.53
	Bacillales	*Planomicrobium*	OTU1704	15	5	10	2.31
JAL-NAL	Rhizobiales	*Methylorubrum*	OTU3111	14	8	6	2.43
	Micrococcales	*Kocuria*	OTU1586	13	4	9	2.91
	Kineosporiales	*Quadrisphaera*	OTU3335	13	8	5	2.95
	Rhodobacterales	*Paracoccus*	OTU1744	13	4	9	1.59
	Rhizobiales	*Methylorubrum*	OTU3325	13	8	5	5.87
	Bacillales	*Planomicrobium*	OTU1704	13	5	8	0.78
	Kineosporiales	*Kineococcus*	OTU1590	13	8	5	1.68
	Rhodobacterales	*Paracoccus*	OTU172	12	9	3	0.77
	Rhizobiales	*Methylorubrum*	OTU3872	12	5	7	1.42
	Acetobacterales	*Roseomonas*	OTU3312	12	7	5	0.80
JSM-NSM	Micrococcales	*Kocuria*	OTU1586	13	4	9	5.59
	Micrococcales	*Microterricola*	OTU3382	13	8	5	1.72
	Micrococcales	*Cellulomonas*	OTU1826	13	4	9	1.31
	Sphingomonadales	*Novosphingobium*	OTU3305	13	8	5	4.90
	Micrococcales	*Microbacterium*	OTU3225	13	8	5	0.86
	Micrococcales	*Arthrobacter*	OTU1655	12	4	8	12.61
	Micrococcales	*Rathayibacter*	OTU1234	12	7	5	0.54
	Sphingomonadales	*Novosphingobium*	OTU3374	12	7	5	1.55
	Sphingomonadales	*Sphingomonas*	OTU3873	12	7	5	2.09
	Bacillales	*Planomicrobium*	OTU1704	12	3	9	4.32
JAM-NAM	Rhizobiales	*Methylorubrum*	OTU3657	12	11	1	2.16
	Propionibacteriales	*Microlunatus*	OTU1665	12	11	1	0.92
	Rhizobiales	*Methylorubrum*	OTU3872	12	11	1	2.67
	Corynebacteriales	*Rhodococcus*	OTU536	12	11	1	1.25
	Sphingomonadales	*Sphingomonas*	OTU2911	10	8	2	1.04
	Rhodobacterales	*Paracoccus*	OTU172	10	10	0	1.37
	Sphingomonadales	*Sphingomonas*	OTU3873	10	10	0	0.89
	Rhizobiales	*Methylorubrum*	OTU854	10	10	0	2.69
	Corynebacteriales	*Mycobacterium*	OTU2313	10	10	0	1.44
	Sphingomonadales	*Sphingomonas*	OTU3459	10	10	0	2.40
JLJ-NLJ	Rhodobacterales	*Paracoccus*	OTU1744	14	8	6	1.31
	Bacillales	*Planomicrobium*	OTU1704	14	7	7	0.55
	Burkholderiales	*Massilia*	OTU3257	14	8	6	0.91
	Rhodobacterales	*Rubellimicrobium*	OTU1100	14	8	6	0.76
	Sphingomonadales	*Sphingomonas*	OTU2911	13	9	4	0.47
	Deinococcales	*Deinococcus*	OTU3885	13	4	9	1.36
	Bacillales	*Bacillus*	OTU1669	13	7	6	0.47
	Kineosporiales	*Quadrisphaera*	OTU3335	11	4	7	0.87
	Micrococcales	*Arthrobacter*	OTU1655	11	6	5	2.23
	Azospirillales	*Skermanella*	OTU2556	11	6	5	0.89

The top 10 phyllosphere epiphytic bacterial or fungal OTUs with the highest degrees were recognized as keystone taxa (**Tables 3**, **4**). The composition of keystone taxa in bacterial or fungal communities varied as a result of plant species and seasonal changes. The majority of associations involving keystone fungal taxa exhibited positive interactions, indicating that the fungal communities were predominantly influenced by positive interactions (**Table 4**). Nevertheless, the interaction patterns within bacterial communities exhibited a certain level of ambiguity, as a considerable number of negative associations were observed (**Table 3**).

**Table 4:** The ten keystone species in phyllospheric epiphytic fungi networks for plant species and seasons.

	Order	Genus	OTU	degree	+	-	Abundance (%)
Jun	Pleosporales	*Paraphoma*	OTU1209	13	13	0	0.39
	Cystobasidiomycetes	*Symmetrospora*	OTU1139	11	11	0	0.11
	Pleosporales	*Neosetophoma*	OTU788	10	10	0	0.20
	Pleosporales	unclassified	OTU1217	9	9	0	0.29
	Hypocreales	*Gibberella*	OTU257	9	9	0	0.60
	Hypocreales	*Alfaria*	OTU562	9	9	0	0.07
	unclassified Basidiomycota	unclassified Basidiomycota	OTU1534	8	8	0	0.15
	Pleosporales	*Paraphoma*	OTU1088	8	8	0	0.15
	Hypocreales	*Gibberella*	OTU547	8	8	0	0.13
	Tremellales	*Vishniacozyma*	OTU14	7	7	0	1.20
Nov	Cystobasidiomycetes	*Symmetrospora*	OTU1139	6	3	3	4.86
	Pleosporales	unclassified	OTU765	6	5	1	0.68
	Sporidiobolales	*Sporidiobolus*	OTU1427	6	3	3	1.20
	Tremellales	unclassified	OTU585	5	1	4	0.53
	Erythrobasidiales	*Erythrobasidium*	OTU305	5	3	2	0.54
	Helotiales	*Articulospora*	OTU618	5	2	3	2.14
	Tremellales	*Saitozyma*	OTU874	4	2	2	0.72
	Tremellales	*Vishniacozyma*	OTU14	4	1	3	1.47
	Tremellales	*Hannaella*	OTU602	4	1	3	1.27
	Tremellales	*Vishniacozyma*	OTU692	4	1	3	0.46
JBC-NBC	Pleosporales	*Leptospora*	OTU616	11	4	7	0.16
	Trichosphaeriales	*Nigrospora*	OTU736	11	10	1	0.16
	Pleosporales	*Torula*	OTU1261	10	5	5	1.15
	Erythrobasidiales	*Erythrobasidium*	OTU117	10	9	1	0.26
	Tremellales	*Cryptococcus*	OTU1563	10	8	2	2.65
	Tremellales	*Hannaella*	OTU1533	10	8	2	0.14
	Tremellales	*Dioszegia*	OTU1560	10	9	1	1.00
	Tremellales	*Hannaella*	OTU1518	10	8	2	0.35
	Tremellales	unclassified	OTU585	10	9	1	0.23
	Agaricales	unclassified	OTU508	9	7	2	0.37
JAL-NAL	Venturiales	*Ochroconis*	OTU644	18	17	1	0.15
	Pleosporales	*Leptospora*	OTU700	18	17	1	0.26
	Pleosporales	*Didymella*	OTU687	15	14	1	0.72
	Pleosporales	*Paraphoma*	OTU1088	15	14	1	0.17
	Pleosporales	*Setophaeosphaeria*	OTU632	15	13	2	1.84
	Capnodiales	*Cercospora*	OTU1236	15	13	2	0.80
	Pleosporales	*Torula*	OTU1261	15	13	2	0.13
	Hypocreales	*Acremonium*	OTU497	14	12	2	1.81
	Agaricostilbales	*Kondoa*	OTU846	14	12	2	0.50
	Tremellales	*Vishniacozyma*	OTU692	14	12	2	0.73
JSM-NSM	Tremellales	*Saitozyma*	OTU748	12	10	2	0.45
	Capnodiales	*Cladosporium*	OTU335	9	1	8	0.41
	Tremellales	*Hannaella*	OTU1528	9	7	2	0.45
	Cystofilobasidiales	*Cystofilobasidium*	OTU824	9	5	4	0.44
	Capnodiales	unclassified	OTU972	8	6	2	2.49
	unclassified Basidiomycota	unclassified	OTU1534	8	4	4	2.58
	Sporidiobolales	*Rhodotorula*	OTU190	8	3	5	0.48
	Tremellales	*Vishniacozyma*	OTU14	8	1	7	0.52
	Dothideales	*Aureobasidium*	OTU792	7	5	2	0.42
	Capnodiales	*Cercospora*	OTU1236	7	5	2	1.61
JAM-NAM	Pleosporales	*Didymella*	OTU1304	14	14	0	0.92
	Tremellales	*Hannaella*	OTU1463	13	10	3	0.25
	Pleosporales	unclassified	OTU1217	13	10	3	0.57
	Pleosporales	*Coniothyrium*	OTU1042	12	11	1	0.63
	Cystobasidiomycetes	*Symmetrospora*	OTU430	12	9	3	3.27
	Agaricales	*Flammulina*	OTU767	12	11	1	0.31
	Pleosporales	*Leptosphaeria*	OTU1119	12	11	1	0.30
	Hypocreales	*Fusarium*	OTU1158	11	11	0	0.64
	Dothideales	*Aureobasidium*	OTU348	11	11	0	0.33
	Pleosporales	*Neosetophoma*	OTU788	10	10	0	0.30
JLJ-NLJ	Sporidiobolales	*Sporidiobolus*	OTU1427	15	14	1	2.04
	Pleosporales	unclassified	OTU1069	14	14	0	0.43
	Tremellales	*Hannaella*	OTU1528	14	14	0	1.43
	Pleosporales	*Setophaeosphaeria*	OTU632	14	14	0	0.20
	Cystobasidiomycetes	*Symmetrospora*	OTU800	13	11	2	0.15
	unclassified Basidiomycota	unclassified Basidiomycota	OTU1534	13	11	2	3.83
	Pleosporales	*Didymella*	OTU687	11	11	0	0.85
	Pleosporales	*Didymella*	OTU1270	11	11	0	0.97
	Pleosporales	*Epicoccum*	OTU1366	11	11	0	1.60
	Erythrobasidiales	*Erythrobasidium*	OTU117	11	11	0	2.08

Unclassified *Arthrobacter* (OTU 1655, *Micrococcales*) was identified as a bacterial keystone taxa in both seasons (**Figures 7A, B**). It accounted for 25.46% and 11.86% of the OTUs of the bacterial network nodes, respectively, among the *Frankiales*, *Micrococcales*, *Rhodobacterales*, and *Sphingomonadales*. These taxa were found among plants in both summer and winter. In contrast, none of the bacterial taxa were found in all five species that formed the inter-seasonal co-occurrence network. However, *Micrococcaceae*, *Rhizobiales*, and *Bacillariophyceae* were present in four different species. Among these, *Micrococcaceae* had the highest representation in the inter-seasonal network nodes in *B. chinense*, *S. miltiorrhiza*, and *L. japonica*, accounting for 9.52%, 22.63%, and 2.23% of the nodal OTUs, respectively. *Rhizobiales* were found to be present in *A. lancea* and *A. membranaceus*, with *A. membranaceus* accounting for the highest percentage of nodes OTU at 9.72%, followed by *A. lancea* at 7.52%. This finding indicates that the presence of Micrococcus taxa and *Rhizobiales* taxa is significant in establishing a bacterial network that connects medicinal plants across different seasons (**Table 3**).

*Pleosporales*, *Tremellales*, and *Cystobasidiomycetes* were identified as significant fungal keystone taxa during both seasons. Among these, *Tremellales* exhibited the highest proportion of OTUs in the summer node, accounting for 1.20%. On the other hand, *Cystobasidiomycetes* displayed the highest proportion of OTUs in the winter node, accounting for 4.86%. In addition, the presence of *Tremellales* was observed in the interseasonal symbiotic network of all five species. The predominant group of OTUs found in *B. chinense* was *Tremellales*, accounting for 4.37% of the total. The predominant fungal taxa in *A. lancea* and *L. japonica* were *Pleosporales*, accounting for 3.12% and 4.05% of the total nodes, respectively. In contrast, *Capnodiales* represented the highest percentage (4.51%) in *S. miltiorrhiza*, while *A. membranaceus* exhibited a prevalence of *Cystobasidiomycetes*, accounting for 2.72% of the total nodes. Thus, various keystone taxa are responsible for connecting inter-seasonal fungal networks among different host plant species (**Table 4**).

For the analysis of bacterial communities, the MCODE algorithm identified one module in the summer and five modules in the winter that exhibited statistical significance (**Supplementary Figures S2A, B**). This suggests that the network associations among plants were more intricate during the winter compared to the summer. In addition, the MCODE analysis predicted a total of 5 modules for *B. chinense*, 5 for *A.lancea*, 4 for *S.miltiorrhiza*, 3 for *A.membranaceus*, and 6 for *L.japonica* across seasons for each respective host plant (**Supplementary Figures S2C–G**). The phyllosphere epiphytic fungal network exhibited two statistically significant modules during the summer and five modules during the winter (**Supplementary Figures S3A, B**). Modules were also identified within the epiphytic fungal network of five different plant species (*B.chinense*, *A.lancea*, *S.miltiorrhiza*, *A.membranaceus*, and *L.japonica*) across multiple seasons. The number of modules observed in each species were as follows: 6 in *B. chinense*, 5 in *A.lancea*, 6 in *S.miltiorrhiza*, 5 in *A.membranaceus*, and 5 in *L.japonica* (**Supplementary Figures S3C–G**). Our findings indicate the presence of intensive networks within phyllosphere epiphytic microbial communities across different seasons in all the medicinal plants examined. Furthermore, the networks of both epiphytic bacterial and fungal communities among plants were found to be more intricate during the winter compared to the summer.

### 3.6 Functional prediction

**Figure 8:**
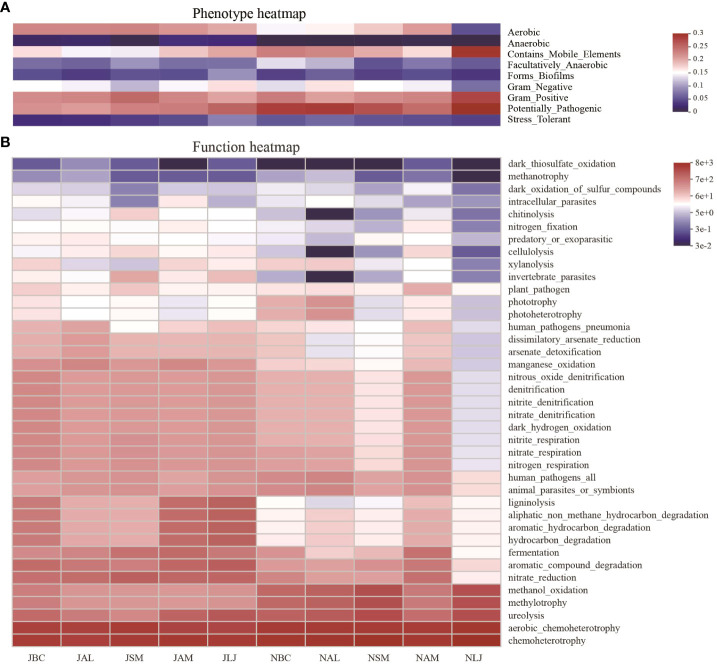
Phenotypic **(A)** and functional prediction **(B)** of phyllospheric epiphytic bacterial communities. J, June-Summer; N, November-Winter; BC, *Bupleurum chinense*; AL, *Atractylodes lancea*; SM, *Salvia miltiorrhiza*; AM, *Astragalus membranaceus*; LJ, *Lonicera japonica*.

The BugBase microbiome analysis tool was employed to forecast phenotypes for the bacterial communities residing on the phyllosphere as epiphytes. The findings demonstrated consistent trends in the phenotypic composition of phyllosphere epiphytic bacterial communities among plants in both seasons (**Figure 8A**). The phenotypic composition of bacterial communities on each host species exhibited notable variations across different seasons. Aerobic, mobile element-containing, Gram-positive, and pathogenic phenotypes were found to be prevalent among phyllosphere bacteria. The Kruskal-Wallis tests showed that mobile element containing and aerobic were phenotypes with significant differences between plants in both summer and winter (**Supplementary Figures S4A, B**). Furthermore, there were significant variations observed in the anaerobic, biofilm forming, facultatively anaerobic, Gram positive, and pathogenic phenotypes among the five host plants during the winter. Meanwhile, the phenotypes of epiphytic bacterial communities exhibited notable seasonal variations in our study (**Supplementary Figure S4C**). Epiphytic bacteria with mobile element containing phenotype were significantly different in abundance between seasons for all five plants, and the abundance of bacteria possessing this phenotype was significantly higher in summer in four plants except in *A. membranaceus*.

A comprehensive analysis was conducted using the FAPROTAX database to predict a total of 39 bacterial ecological functions within the phyllosphere epiphytic bacterial communities. The ecological functions of bacterial communities across different plant species exhibited seasonal variations. Specifically, during the winter season, the bacterial communities of five plant species demonstrated a higher level of function heterogeneity compared to those observed during the summer (**Figure 8B**). The ecological functions of bacterial communities were found to be primarily dominated by chemoheterotrophy, accounting for 23.96% to 38.09% of the overall abundance of the bacterial community. Additionally, aerobic chemoheterotrophy (RA=17.11%-28.86%). In accordance with the results obtained from the heat map analysis, it was observed that the functions of aerobic chemoheterotrophy and chemoheterotrophy were the predominant features in the composition of epiphytic bacterial communities. The results of the Kruskal-Wallis tests revealed that eight out of the nine dominant functions displayed statistically significant variations in abundance among plants during the winter season. However, no significant differences were observed in the abundance of these functions during the summer (**Supplementary Figures S5A, B**).

Seasonal comparisons on abundances of bacteria possessing the top 9 dominant functions indicated that certain types of ecological function showed accordant patterns in seasonal changes in all five host species, and some with statistical significance (**Supplementary Figure S5C**). For example, there were more bacteria with nitrate reduction in summer than winter for all five host species, and the differences were significant in four plants except AM. The study revealed a higher abundance of chemoheterotrophic bacteria during the winter season in five plant species, specifically *B. chinense*, *A.lancea*, and *L.japonica*. These differences in abundance were statistically significant. The prevalence of methanol oxidation bacteria exhibited a consistent increase during the winter season, with significantly higher levels observed in *A.lancea*, *S.miltiorrhiza*, and *L.japonica*.

**Figure 9:**
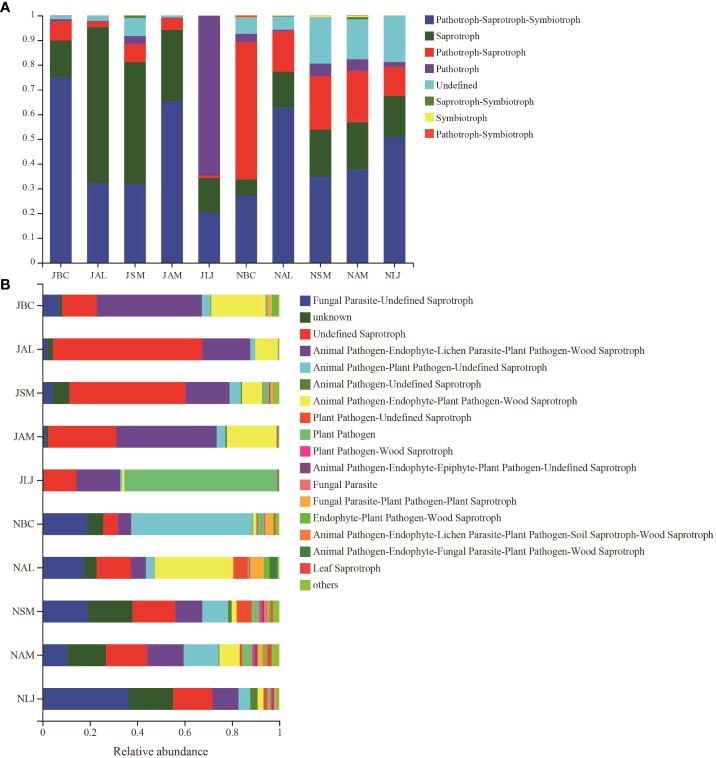
Functional prediction of phyllosphere epiphytic fungal community base on FUNGuild database. **(A)** Basic functional classification of different epigenetic fungi; **(B)** multiple detailed functional classification of different epigenetic fungi J, June- Summer; N, November-Winter; BC, *Bupleurum chinense*; AL, *Atractylodes lancea*; SM, *Salvia miltiorrhiza*; AM, *Astragalus membranaceus*; LJ, *Lonicera japonica*.

Based on their mode of nutrition, the epiphytic fungi were categorized into three primary trophic groups as per the FUNGuild prediction: pathotrophs (including phagotrophic fungi phagotrophs), symbiotrophs, and saprophytes. The findings indicated that during the summer, the epiphytic fungal community in LM was predominantly comprised of pathotrophs (RA=64.63%) and pathotroph-saprotroph-symbiotrophs (RA=20.42%). In contrast, the fungal communities in the other four plants during the summer were primarily saprotrophs (RA=14.79%-63.08%) and pathotroph-saprotroph-symbiotrophs(RA=31.83%-75.06%). The pathotroph-saprotroph-symbiotrophs (RA=27.23%-62.70%) was as the dominant functional group of epiphytic fungi in winter. Notably, there was a significant decrease in the abundance of fungal saprotrophs (RA=6.43%-19.06%) and a corresponding increase in pathotroph-saprotrophs (RA= 11.80%-55.62%) across all host species during the winter, as compared to the summer communities (**Figure 9A**).

The epiphytic fungi were further classified into twelve distinct functional guilds. The findings of the study revealed that the dominant types of epiphytic fungi on *L. japonica* during the summer were primarily plant pathogens (RA = 64.62%). Additionally, a significant proportion of the fungi belonged to the categories of Animal Pathogen-Endophyte-Lichen Parasite-Plant Pathogen-Wood Saprotroph (RA=18.44%) and Undefined Saprotroph (RA=13.74%). On the other hand, the epiphytic fungi found on the other four plants were predominantly categorized as Undefined Saprotroph (RA=14.64%-63.05%), Animal Pathogen-Endophyte-Lichen Parasite-Plant Pathogen-Wood Saprotroph (RA=18.63%-44.35%), and Animal Pathogen-Endophyte-Plant Pathogen-Wood Saprotroph (RA=8.58%-22.95%). The diversity of ecological functions performed by epiphytic fungi on five plant species was found to be higher during the winter season. Specifically, there was an increase in the presence of Animal Pathogen-Plant Pathogen-Undefined Saprotroph fungi (RA=3.58%-51.27%) and Fungal Parasite-Undefined Saprotroph fungi (RA =10.64%-36.42%) compared to the summer. Conversely, there was a decrease in the abundance of Undefined Saprotroph fungi (RA=6.03%-18.02%) and Animal Pathogen-Endophyte-Lichen Parasite-Plant Pathogen-Wood Saprotroph fungi (RA=5.73%-15.36%).Animal Pathogen-Endophyte-Plant Pathogen-Wood Saprotroph fungi exhibited a decline from summer to winter in all four species (RA=1.40%-8.61%), with the exception of *A. lancea* (RA=33.18%) (**Figure 9B**).

## 4 Discussion

### 4.1 Taxonomic composition of community

In this study, the dominant taxa in the epiphytic bacterial communities of medicinal plants were Actinobacteriota and Proteobacteria. This finding is in line with previous research that investigated phyllosphere epiphytic bacteria using both culture-dependent and -independent approaches.The Actinobacteriota and Proteobacteria were found to be the most prevalent in all of the examined locations. However, their abundance was observed to be higher in 2014 as compared to 2016. In the year 2014, the Actinobacteriota constituted 93.4% and 86.8% of the epiphytic bacterial communities found on plants in rural and urban areas, respectively (Espenshade et al., 2019). Leff et al. (2015) proposed that the elevated rates of reproduction observed in numerous members of the Proteobacteria phylum are the primary factor contributing to the substantial proportion of isolates from this group. Meanwhile, the Firmicutes have been identified as a dominant bacterial group during the summer. This finding is consistent with a previous study by Wei et al. (2022), which reported Firmicutes as the dominant group of phyllosphere epiphytic bacteria. Unlike fungi, bacteria are unable to penetrate the cuticle of plant tissues through mycelium. However, Firmicutes, which are nitrogen-fixing bacteria, are capable of supplementing nitrogen acquisition and adapting to epiphytic niches (Zehr et al., 2003). Furthermore, Bacteroidota, which are also the prevailing phylum found in marine macrophytes, constitute a relatively significant portion of the bacterial community that inhabits winter epiphytic environments (Chen et al., 2022). Bacteroidota bacteria play a crucial role in the degradation of biopolymers, facilitating the growth of colonizing bacteria by creating an aerobic environment within the surface biofilm (Dang et al., 2011). At the order level, *Micrococcales* is a main constituent group of epiphytic bacteria, which has not been reported for phyllosphere epiphytic microorganisms to our knowledge. *Micrococcales* played an important role in networks of epiphytic bacterial communities across plants and across seasons. The ability to degrade biological macromolecules (e.g. cellulose and lignin) may account for the dominance of *Micrococcales* (Liu et al., 2020). The genus *Curtobacterium* has been identified as a significant pathogenic bacterium in economically important crops (Evseev et al., 2022). However, we have identified an unclassified member of the genus *Curtobacterium* (OTU 3394) present on all five medicinal plants during both seasons. The investigation of the interaction between taxa and host plants, as well as the potential ecological consequences of their presence, requires further examination.

The epiphytic fungal community was dominated by *Dothideomycetes* (Ascomycota) and *Tremellomycetes* (Basidiomycota), which is consistent with findings from previous studies conducted on both tropical and temperate plants (Coleman-Derr et al., 2016; Yao et al., 2019; Bao et al., 2022). The *Dothideomycetes* is recognized as the largest and most diverse in terms of ecological and functional characteristics (Haridas et al., 2020; Hongsanan et al., 2020). This group encompasses various species that are known to be pathogens of both humans and plants, as well as endophytes and epiphytes. In addition, *Dothideomyetes* has been widely documented as one of the important taxa related to leaves (Qian et al., 2018; Xiong et al., 2021). *Pleosporales* represents the most extensive order within the class *Dothideomycetes* (Yu et al., 2022). Its constituents exhibit a wide range of ecological roles, including epiphytic, endophytic, and parasitic associations with various host plants (Mapook et al., 2016). *Pleosporales* emerges as a prominent fungal order within the epiphytic fungal community in our study. It encompasses keystone taxa that exhibit the highest abundance within the epiphytic fungal networks of the five medicinal plants under investigation. *Tremellales* exhibited a preference for cold seasons in our study. Taxa belonging to *Tremellales* were identified as keystone taxa in all five medicinal plant species within the epiphytic fungal networks, regardless of the season. The concept of keystone species has been proposed to have a significant impact on the stabilization of microbial communities (Vetrovsky et al., 2020). Our findings indicate that the presence of *Pleosporales* and *Tremellales* taxa significantly contribute to the preservation of network structure and stability within epiphytic fungal communities on medicinal plants in agroecosystems.

### 4.2 Epiphytic bacterial and fungal community construction in phyllosphere

Plants exert a filtering influence on the microbial communities they are associated with (Bringel and Couee, 2015), and the species of the host plant play a significant role in shaping the compositions of these communities (Kim et al., 2012; Yao et al., 2019). Alpha diversity indices of epiphytic bacteria and fungi in this study showed significant differences among all five plant species. Šigutová et al. (2023) demonstrated that the diversity of bacteria was significantly impacted by the species of the host plant, whereas the composition of the fungal community was more strongly influenced by the host species. Bodenhausen et al. (2014) The study found that the host alleles with the greatest influence on the microbiota were lacs2 and pec1, compared to wild-type Arabidopsis. These mutations affect the formation of the cuticle, leading to a significant increase in bacterial abundance, suggesting that different bacteria can benefit from the modified cuticle to varying degrees. In addition, ein2, which is involved in ethylene signaling, was found to be a major host factor regulating the composition of the epigenetic microbial community. In a study conducted by Kang et al. (2022), the diversity indices and community structure of epiphytic fungi in the phyllosphere of bamboo were examined during the spring and autumn seasons. The results revealed significant differences in both the diversity indices and community structure among different bamboo species as well as between the two seasons. Li M. et al. (2022) found that phyllosphere fungal communities of subtropical trees varied with host species identity and seasonality, and that host species identity had a greater effect on phyllosphere fungal community assembly compared to seasonality. The findings from the NMDS and PERMANOVA analyses revealed significant variations in the communities of phyllosphere epiphytic microorganisms of medicinal plants across different plant species and seasons. Additionally, the ANOSIM analysis demonstrated that the phyllosphere epiphytic microorganisms differed between species, plants, and seasons. The dissimilarities in leaf hairiness among plant species were found to be responsible for the variations in phyllosphere epiphytic bacterial communities. Moreover, the level of hairiness indirectly influenced the contact area and habitat of microorganisms with plants, thereby impacting the diversity and structure of phyllosphere microbial communities (Bai et al., 2022).

Previous research has also associated seasonal variations with alterations in the composition of the epiphytic microbial community (Jackson and Denney, 2011; Warshan et al., 2016). We observed that the alpha-diversity indices of bacterial and fungal communities exhibited a greater degree of variation between seasons as opposed to variations between plants. Seasonal variations exhibited significant impacts on the richness of epiphytic bacteria and fungi, aligning with findings from previous research (Vokou et al., 2019; Wei et al., 2022). In the current study, it was observed that there was a higher richness of bacterial OTUs during the summer compared to the winter. Conversely, a greater diversity of fungal OTUs was found during the winter as opposed to the summer. Šigutová et al. (Šigutová et al., 2023) conducted a study to investigate the impact of season on the composition of epiphytic bacterial communities. The results revealed that there were significant variations in the bacterial communities between different months, with the most pronounced differences observed between April and the other months. Zhang et al. (2022) employed NMDS plots utilizing Bray-Curtis distances and conducted PERMANOVA analysis to characterize the bacterial community structure across all samples of Medicago sativa. The study revealed notable differences between samples in each season, indicating significant distinctions. Zheng (2011) discovered that the diversity of the microbial community in the phyllosphere of Pinus exhibited the highest diversity during autumn, followed by summer and spring. In contrast, Thompson et al. (1993) demonstrated that the diversity of the epiphytic fungal community in the phyllosphere of Beta vulgaris was lower in spring compared to autumn. The variation in leaf characteristics and environmental factors between young and old leaves, along with the influence of host plant species, play crucial roles in shaping leaf-associated communities and account for the observed differences (Šigutová et al., 2023). Overall, our study provides confirmation of significant seasonal fluctuations in the composition of phyllosphere epiphytic bacteria and fungi (Jumpponen and Jones, 2010; Gomes et al., 2018; Postiglione et al., 2022).

In this study, it was observed that both host species and season played a significant role in influencing the presence of epiphytic microorganisms. However, it is worth noting that the limitations of the planting site may have affected the season’s impact on interleaf epiphytic microorganisms at a smaller habitat scale, resulting in a weaker host selectivity.

### 4.3 Symbiotic patterns of epiphytic bacterial and fungal communities

Network analysis, utilizing correlation tests, has been increasingly employed in recent years to enhance comprehension of the interactions among community members within microbial communities (Qian et al., 2018; Xiong et al., 2021). In our study, the co-occurring networks formed by the fungal or bacterial communities continued to exhibit seasonal variations. For instance, during the summer, there was a decrease in the number of interactions observed in the bacterial network compared to the winter. Conversely, the opposite trend was observed for fungi, with an increase in the number of interactions during the summer. Liu et al. (2022) elucidated substantial fluctuations in microbial networks across distinct seasons within a comprehensive investigation of lake ecosystems. The researchers discovered that the autumn season displayed the highest level of complexity and resilience in the network. This conclusion was ascribed to the phenomenon of environmental filtering and its associated interspecies interactions, wherein certain taxonomic groups exhibited distinct characteristics specific to different seasons. Interestingly, it was also found that bacteria and fungi exhibited a higher occurrence of interspecies interactions and positive correlations when there was a decrease in overall diversity.

The networks of each plant exhibited a higher number of connections compared to the networks of each season. This suggests that the interactions between epiphytic bacteria and fungi primarily took place among individuals of the same host species, rather than between individuals of different host species. For each plant species examined, there was a predominantly positive correlation between the types of bacterial and fungal network interactions observed in *B.chinense*, *S.miltiorrhiza*, and *A.membranaceus*. This finding suggests that mutualistic symbiosis between microorganisms plays a dominant role in these plants. On the other hand, there were numerous negative correlations observed in the bacterial network interaction types for *A.lancea* and *L. japonica*. In contrast, fungi exhibited a predominance of positive correlations. These findings suggest that in these plants, there exist not only mutually beneficial symbiotic relationships between microorganisms, but also frequent negative interactions, including antagonism, competition, and parasitism.

The stability of a microbial community is contingent upon its modular structure and the presence of keystone taxa (Liu et al., 2022). In the present study, the formation of modules was frequently observed within networks comprising bacterial and fungal communities. We observed that the network structure of winter networks exhibited a higher degree of modularity compared to summer networks. Additionally, we noted a decrease in connectivity among fungal modules during the winter. This observation implies that the selection of connections between plant hosts may exhibit a stronger preference for a specific season. As modular structures have been found to provide protection to communities against secondary extinctions that occur after disturbances, they also enhance the stability of the entire network (Stouffer and Bascompte, 2011). In the context of inter-seasonal networks, the level of modularity observed in individual plants was higher compared to inter-plant networks. This finding suggests that epiphytic communities, which consist of different periods of the same host, are more resilient to secondary species extinctions caused by disturbances compared to communities with different hosts within the same period. We conducted an investigation into the impact of host species and seasonal fluctuations on the stability of epiphytic communities. Our findings revealed that the majority of keystone taxa, characterized by high nodes, were species with low relative abundance. Conversely, community members with high abundance displayed limited or no mutualistic relationships. This observation suggests that the dynamics of microbial communities are primarily influenced by infrequent taxa with low population sizes.

### 4.4 Ecological functions of epiphytic bacterial and fungal communities

Any characteristic that is associated with the growth, reproduction, or survival of a plant and has the potential to influence its fitness is commonly referred to as a functional trait (Violle et al., 2007). The plant microbiome is proposed to be a functional characteristic of plants, as the diversity of bacterial communities on leaves has been found to be correlated with host growth and mortality rates (Kembel et al., 2014). In the current study, an examination of bacterial populations indicated that the relative abundance of Gram-negative bacteria remained stable and dominant across different seasons. The proportion of Gram-positive bacteria exhibited a peak during the summer months and subsequently experienced a gradual decline throughout the winter. The presence of an outer lipid membrane in Gram-negative bacteria can be attributed to this phenomenon, which presents a challenge for penetration. Consequently, Gram-negative bacteria exhibit an additional layer of protection that is not present in Gram-positive bacteria. Additionally, chemoenergetic heterotrophic bacteria serve a pivotal function as the principal component of epiphytic bacteria. These bacteria employ plant organisms as a source of carbon and energy to synthesize their own organic compounds. Phyllosphere epiphytic fungi predominantly demonstrate saprophytic trophic characteristics, leveraging their saprophytic nature to break down plant epidermal cells and obtain the necessary nutrient resources necessary for their sustenance. Based on the classification of trophic types into 13 functions, the high occurrence of indeterminate saprophytic fungi implies that the relationship between epiphytic fungi and the plant organism is predominantly parasitic.

In the present study, it was discovered that diverse epiphytic microbial communities thrive in even the most challenging phyllosphere environments. Furthermore, the composition and species diversity of both epiphytic bacterial and fungal communities exhibited significant variations across seasons and among different medicinal plant species. The influence of season on the composition of epiphytic microbial communities in the phyllosphere of medicinal plants is more pronounced than its impact on the host species. The presence of epiphytic microbes in the phyllosphere is dependent on a consistent microbiota across different plant species during various seasons. Additionally, the association between different plant species during different seasons is characterized by distinct microbiota. Lemanceau et al. (2017) discovered that various plant species possess distinct microbial communities. Within the context of functional redundancy in plant-associated microbial communities, there is a distinct subset known as the “core microbiome.” This core microbiome plays a critical role in maintaining the health of the host plant by carrying essential genes. The dissimilarities observed in the epiphytic microbial communities of medicinal plants were more pronounced during the winter compared to the summer. The significant correlation between the host plant’s significance in the phytosphere and the microbial community of the phytosphere underscores the necessity for a thorough examination of the interactions between the host plant and the epiphytic microbial community of the phytosphere. This study also establishes a theoretical foundation for the potential application of foliar fungicides in medicinal contexts.

## Data availability statement

The original contributions presented in the study are included in the article/**Supplementary Material**, further inquiries can be directed to the corresponding authors.

## Author contributions

CH: Conceptualization, Formal Analysis, Investigation, Methodology, Resources, Writing – original draft, Writing – review & editing. MZ: Formal Analysis, Investigation, Writing – review & editing. XL: Conceptualization, Funding acquisition, Project administration, Supervision, Writing – review & editing. XH: Conceptualization, Methodology, Project administration, Supervision, Writing – review & editing.
