# Supplementary material for: Seasonal dynamics of phyllosphere epiphytic microbial communities of medicinal plants in farmland environment
Source: Front Plant Sci. 2024 Jan 4;14:1328586. doi: 10.3389/fpls.2023.1328586 (PMC10794659; doi:10.3389/fpls.2023.1328586)
Supplement: Supplementary file 1 [file Image_1.pdf]

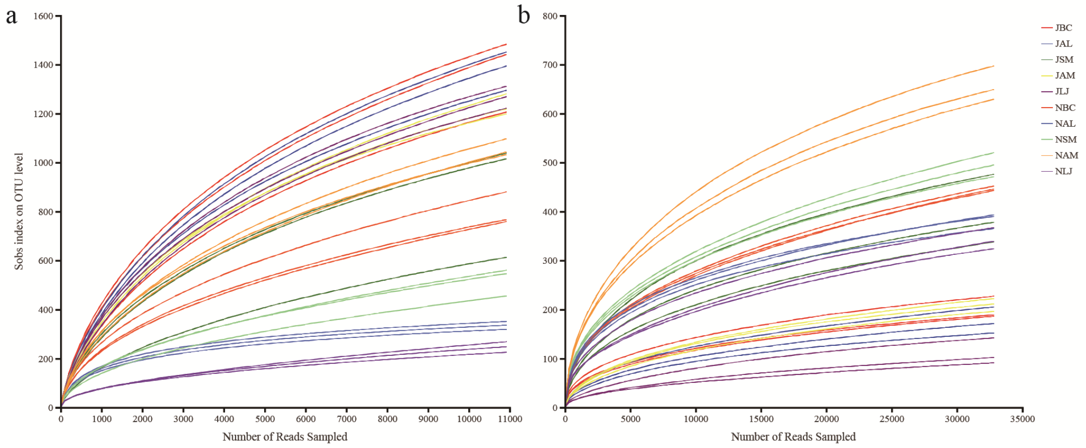

Fig.S1 Rarefaction curves for phyllospheric epiphytic bacterial and fungal operational taxonomic units (OTUs).
Note: a, epiphytic bacteria; b, epiphytic fungi; J, June; D, November; BC, *Bupleurum chinense*; AL, *Atractylodes lancea*; SM, *Salvia miltiorrhiza*; AM, *Astragalus membranaceus*; LJ, *Lonicera japonica*.

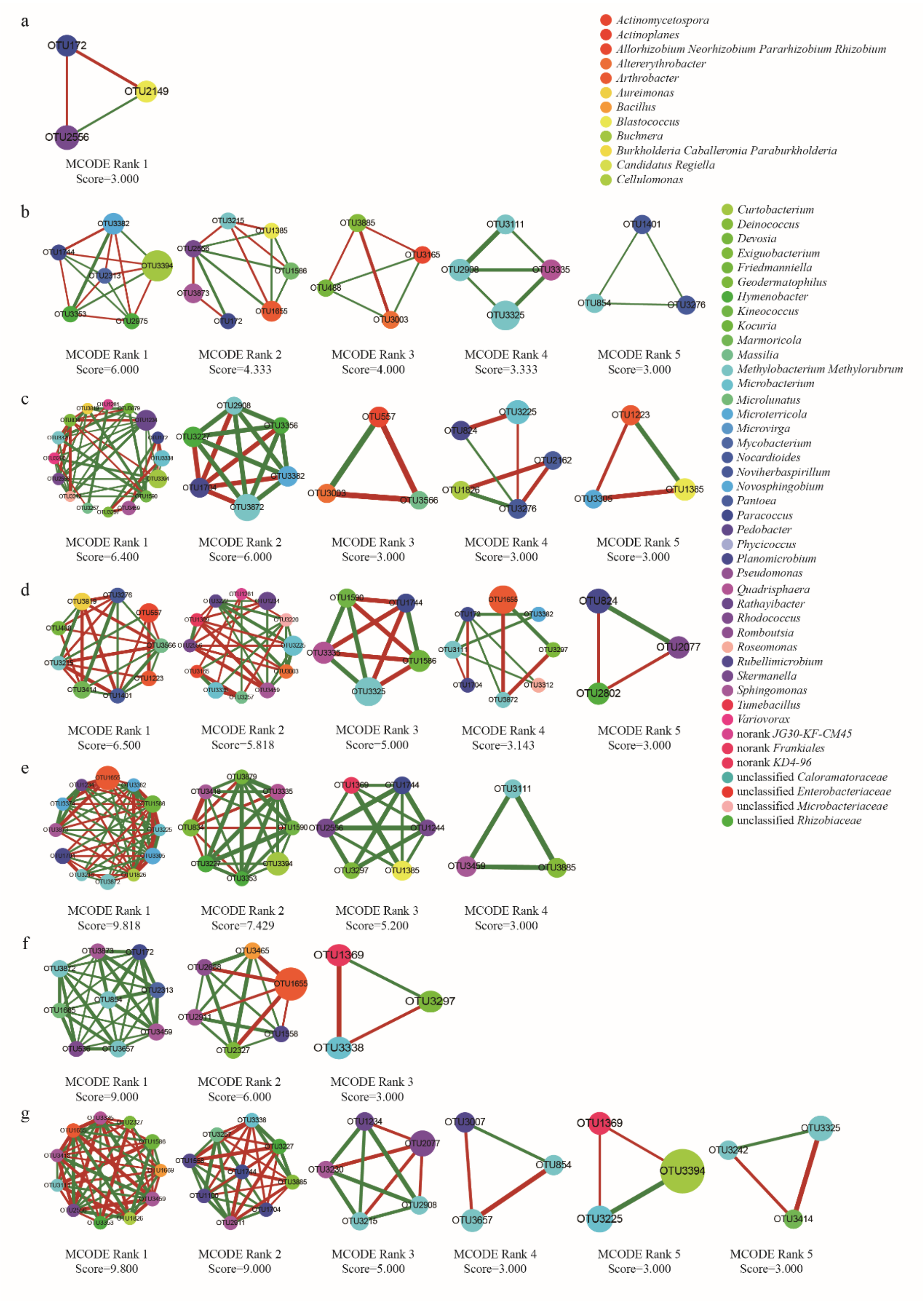

Fig.S2 MCODE analysis showing the modular groups of highly interconnected nodes in networks.
Note: a, June sample; b, November sample; c-g, BC, AL, SM, AM and LJ in that order.

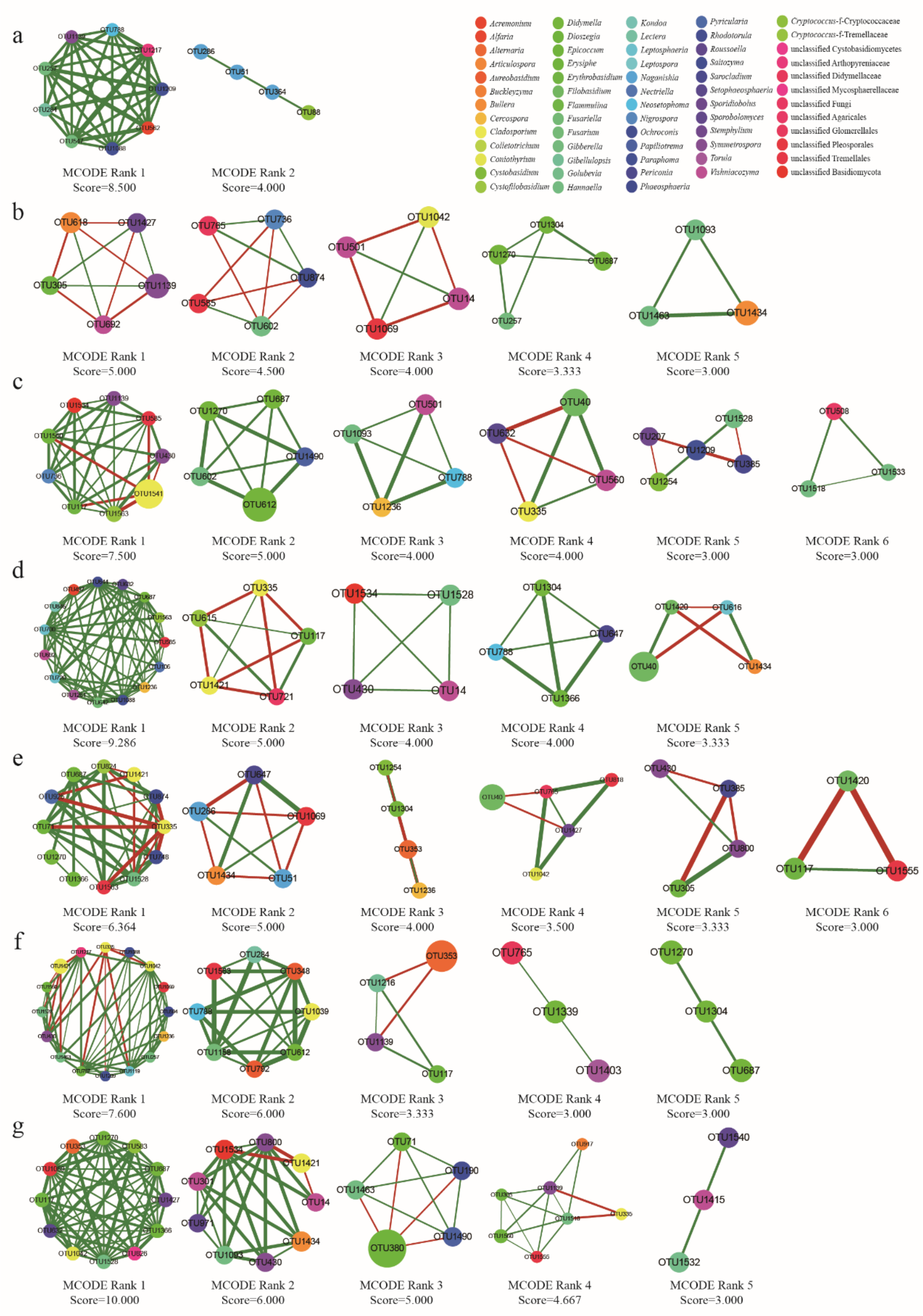

Fig. S3 MCODE analysis showing the modular groups of highly interconnected nodes in networks.

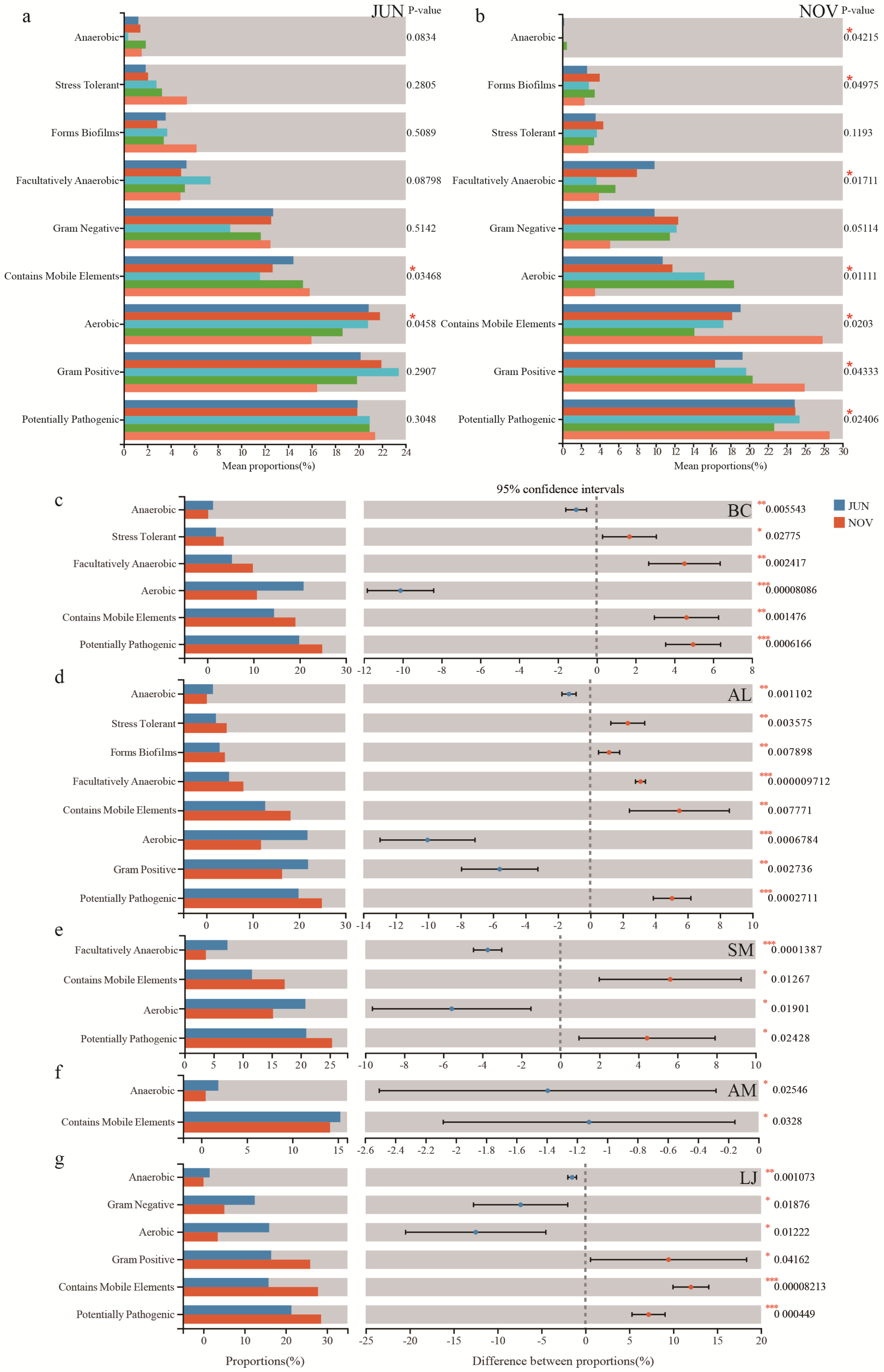

Fig.S4 The influence of seasons(a,b)and plant species(c-g) on the phenotypic prediction of phyllospheric epiphytic bacterial communities.BC:*Bupleurum chinense*; AL:*Atractylodes lancea*; SM:*Salvia miltiorrhiza*; AM:*Astragalus membranaceus*; LJ:*Lonicera japonica.*

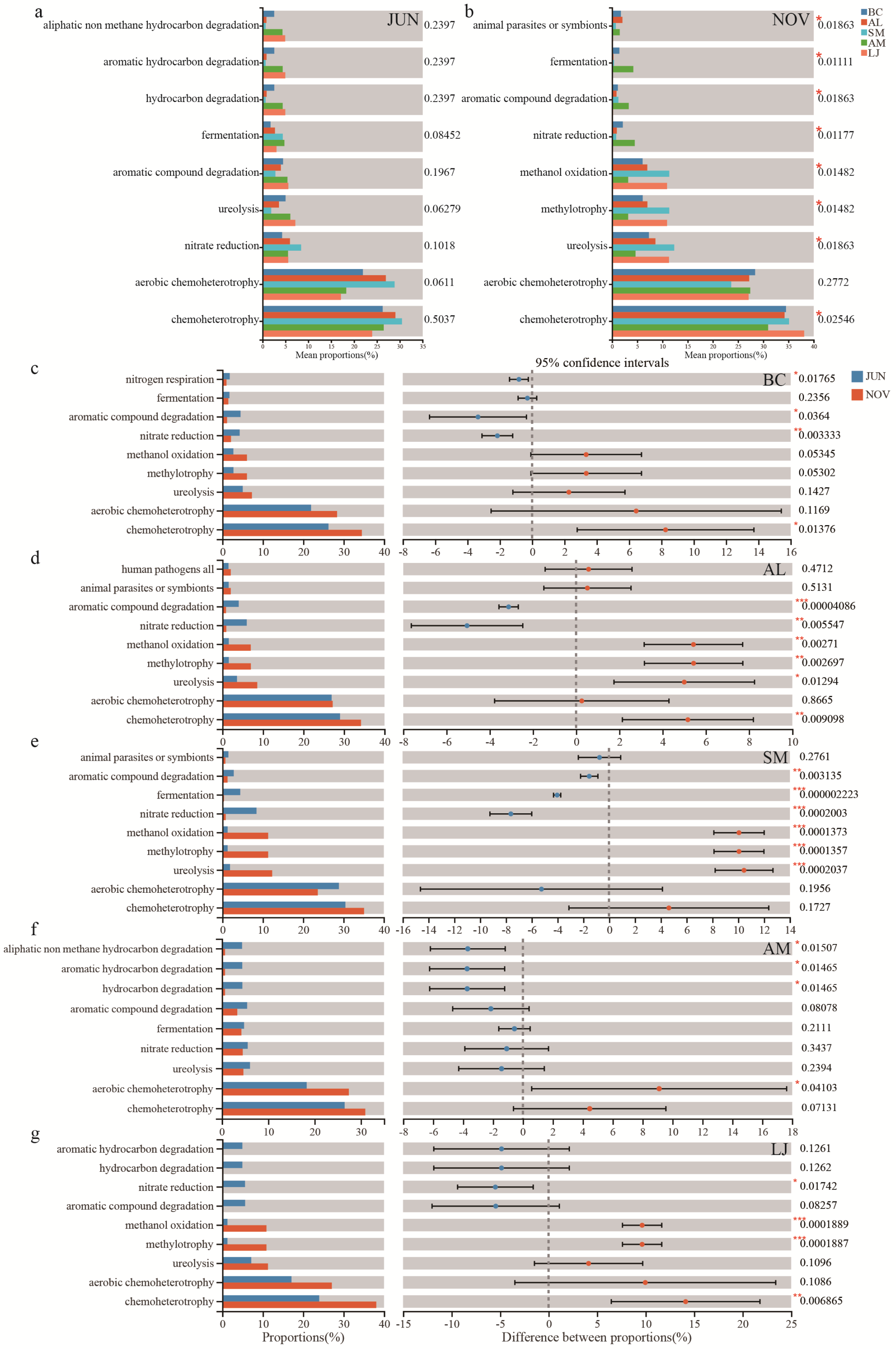

Fig.S5 The influence of seasons(a,b) and plant species(c-g) on the ecological functions prediction of phyllospheric epiphytic bacterial communities.BC:*Bupleurum chinense*; AL:*Atractylodes lancea*; SM:*Salvia miltiorrhiza*; AM:*Astragalus membranaceus*; LJ:*Lonicera japonica*.

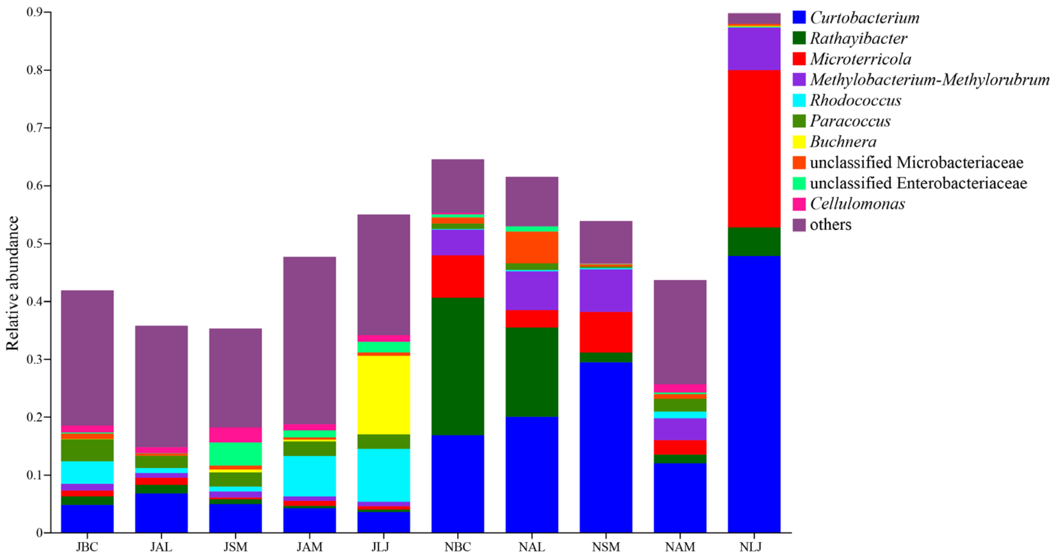

Fig. S6 Species-phenotypic contribution to the function of the epiphytic bacterium Cotains Mobile Elements.J: June-Summer; N: November-Winter; BC: *Bupleurum chinense*; AL: *Atractylodes lancea*; SM: *Salvia miltiorrhiza*; AM: *Astragalus membranaceu*s; LJ: *Lonicera japonica*.
